# Brain effective connectivity and functional connectivity as markers of lifespan vascular exposures in middle-aged adults: The Bogalusa Heart Study

**DOI:** 10.3389/fnagi.2023.1110434

**Published:** 2023-03-14

**Authors:** Kai-Cheng Chuang, Sreekrishna Ramakrishnapillai, Kaitlyn Madden, Julia St Amant, Kevin McKlveen, Kathryn Gwizdala, Ramasudhakar Dhullipudi, Lydia Bazzano, Owen Carmichael

**Affiliations:** ^1^Department of Physics & Astronomy, Louisiana State University, Baton Rouge, LA, United States; ^2^Pennington Biomedical Research Center, Baton Rouge, LA, United States; ^3^Department of Electrical and Computer Engineering, Louisiana State University, Baton Rouge, LA, United States; ^4^Department of Epidemiology, Tulane University School of Public Health and Tropical Medicine, New Orleans, LA, United States

**Keywords:** effective connectivity, functional connectivity, fMRI, aging, cardiovascular risk

## Abstract

**Introduction:**

Effective connectivity (EC), the causal influence that functional activity in a source brain location exerts over functional activity in a target brain location, has the potential to provide different information about brain network dynamics than functional connectivity (FC), which quantifies activity synchrony between locations. However, head-to-head comparisons between EC and FC from either task-based or resting-state functional MRI (fMRI) data are rare, especially in terms of how they associate with salient aspects of brain health.

**Methods:**

In this study, 100 cognitively-healthy participants in the Bogalusa Heart Study aged 54.2 ± 4.3years completed Stroop task-based fMRI, resting-state fMRI. EC and FC among 24 regions of interest (ROIs) previously identified as involved in Stroop task execution (EC-task and FC-task) and among 33 default mode network ROIs (EC-rest and FC-rest) were calculated from task-based and resting-state fMRI using deep stacking networks and Pearson correlation. The EC and FC measures were thresholded to generate directed and undirected graphs, from which standard graph metrics were calculated. Linear regression models related graph metrics to demographic, cardiometabolic risk factors, and cognitive function measures.

**Results:**

Women and whites (compared to men and African Americans) had better EC-task metrics, and better EC-task metrics associated with lower blood pressure, white matter hyperintensity volume, and higher vocabulary score (maximum value of *p* = 0.043). Women had better FC-task metrics, and better FC-task metrics associated with APOE-ε4 3–3 genotype and better hemoglobin-A1c, white matter hyperintensity volume and digit span backwards score (maximum value of *p* = 0.047). Better EC rest metrics associated with lower age, non-drinker status, and better BMI, white matter hyperintensity volume, logical memory II total score, and word reading score (maximum value of *p* = 0.044). Women and non-drinkers had better FC-rest metrics (value of *p* = 0.004).

**Discussion:**

In a diverse, cognitively healthy, middle-aged community sample, EC and FC based graph metrics from task-based fMRI data, and EC based graph metrics from resting-state fMRI data, were associated with recognized indicators of brain health in differing ways. Future studies of brain health should consider taking both task-based and resting-state fMRI scans and measuring both EC and FC analyses to get a more complete picture of functional networks relevant to brain health.

## 1. Introduction

Functional relationships between distinct brain regions in distributed networks have become essential to our understanding of the neural substrates of cognitive function and how they change over the course of development, maturation, aging, and disease progression (Kregel and Zhang, 2007; Eyler et al., 2011; Friston et al., 2013; Dennis and Thompson, 2014). These inter-regional functional relationships, including those derived from functional magnetic resonance imaging (fMRI) data, attempt to go beyond traditional task activation analyses by capturing the dynamics of information flow within the distributed networks (Fellows et al., 2005; Stevens, 2009; Friston, 2011). Most fMRI studies to date have formulated inter-regional functional relationships in terms of signal synchrony (functional connectivity, FC). FC makes no attempt to identify asymmetric relationships between regions, for example relationships wherein the fMRI signal in one region influences the fMRI signal occurring later on in another region. Because FC relationships are symmetric in this way, they are naturally represented using undirected graphs where nodes represent brain regions and edges are drawn between regions with high levels of FC. These graphs have led to new observations about how the brain changes over the course of child development (Bitan et al., 2007; Fair et al., 2010; Jolles et al., 2011; Morken et al., 2017), various brain diseases (Achard and Bullmore, 2007; Fox and Greicius, 2010; Lynall et al., 2010; Van Den Heuvel and Pol, 2010; Gao and Wu, 2016; Geng et al., 2018; Cao et al., 2020), and drug treatment (Wong and Stevens, 2012; Hutcheson et al., 2015; Sarpal et al., 2016; Vai et al., 2016; Cao et al., 2020), as well as how brain functioning relates to cognitive functioning (Supekar et al., 2008; Brier et al., 2014; Archer et al., 2016; Contreras et al., 2020; Sun et al., 2020; Zheng et al., 2021). Several studies have used these graph metrics to suggest that there are aberrant FC patterns in aging (Achard and Bullmore, 2007; Andrews-Hanna et al., 2007; Damoiseaux et al., 2008; Meunier et al., 2009; Mayer et al., 2011; Wu et al., 2011), cognitive impairment (Wang et al., 2006; Allen et al., 2007; Zhang et al., 2009; Sheline and Raichle, 2013), and cardiometabolic disease (Friston et al., 1993; Carnevale et al., 2020). Therefore, methods for quantifying network-level brain functional relationships are currently of intense research interest (Goebel et al., 2003; Sporns, 2007; Deshpande et al., 2009; Liao et al., 2009; Zhou et al., 2011; Nauta et al., 2019; Li et al., 2020; Ambrosi et al., 2021).

A much smaller number of fMRI studies have assessed effective connectivity (EC) between brain regions–the causal influence that functional activity in a source region exerts over functional activity in a target region (Friston, 2011; Friston et al., 2013). EC fundamentally differs from FC as it focuses on more complex and asymmetric relationships between brain regions (Horwitz et al., 2005; Stevens, 2009; Friston, 2011; Gürcan, 2014). These relationships may be either excitatory or inhibitory in nature (Wilson and Cowan, 1972; Tagamets and Horwitz, 1998; Horwitz et al., 2005). Because there is an inherent asymmetry between source and target regions, source-target relationships are naturally represented *via* directed graphs. EC characteristics have been calculated in clinical conditions of interest such as aging (Hinault et al., 2019), cognitive impairment (Luo et al., 2019; Sun et al., 2020), and cardiometabolic disease (Chand et al., 2017).

To our knowledge there have been no head-to-head comparisons of EC and FC in terms of how they associate with factors relevant to various aspects of brain health in a healthy middle-aged cohort. Previous studies including both FC and EC analysis have suggested that EC may be superior to FC for discriminating between brain disease groups, such as stroke patients with differing prognoses (Geng et al., 2018; Adhikari et al., 2021). Others have compared EC and FC patterns that emerge during execution of certain cognitive tasks (Parhizi et al., 2018; Silva et al., 2019). Additional studies have identified different patterns of age-related differences between EC and FC as well as differences among young adults of differing APOE genotypes (Archer et al., 2016; Zheng et al., 2021) To our knowledge, none of these prior studies have compared EC and FC in terms of how they relate to a multi-faceted array of prominent risk factors for late-life cognitive decline among cognitively healthy middle aged adults. In addition, none of these prior methods utilized an EC method that was both *nonlinear* (modeling nonlinear relationships between signals in source and target regions) and *conditional* (accounting for the effects of other regions on the target when modeling source-target relationships). Nonlinear source-target relationships are important to capture because they are believed to represent common cases in neuroscience (Aertsen et al., 1989; Buxton et al., 2004; Grosmark and Buzsáki, 2016), while conditional modeling is important because it reduces the potential for identifying spurious source-target relationships driven by a separate, common source (Chen et al., 2004; Zhou et al., 2009a,b). To address these limitations, we used a novel machine learning based method (Chuang et al., 2021, 2022) to assess nonlinear and conditional EC.

An additional limitation in the literature is that the vast majority of FC and EC analyses have been applied to resting-state rather than task-based fMRI data. Exceptions to this rule have been analyses of differences in FC during task performance between clinically-defined groups (Dennis et al., 2010), age-related changes in task-based fMRI EC (Archer et al., 2016; Hinault et al., 2019), and associations between task-based FC and cognitive function (Koshino et al., 2005; Barch et al., 2013; Monti et al., 2014; Jiang et al., 2020). To our knowledge, only two papers to date have directly compared task-based to resting-state EC, with suggestions that EC information derived from task-based fMRI is richer than corresponding data derived from resting-state fMRI (Archer et al., 2016; Voigt et al., 2021). For this reason, we compared EC and FC measures between resting-state and task-based data.

In this study, we conducted a head-to-head comparison between EC and FC graph metrics derived from task-based and resting-state fMRI in terms of how they correlated with known risk factors for late-life brain health as well as measures of cognitive function in a healthy middle-aged cohort. We used a standard FC method and a state-of-the-art EC method to generate undirected and directed graph representations of individual interregional functional relationships. Metrics derived from these graphs were then evaluated in terms of their associations with demographic, cardiometabolic, and cognitive measures from a middle-aged epidemiological sample.

## 2. Materials and methods

### 2.1. Study participants

The Bogalusa Heart Study began in 1973 as a community-based cohort study of atherosclerosis and risk factors for cardiovascular disease in a Black and White population of children in a rural town in southeastern Louisiana (Berenson, 2001). Participants with a history of stroke or TIA were excluded from the analysis in this study. At the end, 100 participants completed a 3 T brain MRI at Pennington Biomedical Research Center, as well as cardiometabolic measurements and cognitive tests at the Bogalusa Heart Study clinic in Bogalusa, Louisiana (Table 1). Participants in this study provided informed consent. The study was overseen by the Institutional Review Board of Pennington Biomedical Research Center. All Bogalusa Heart Study data may be made available following an approval process through the Bogalusa Heart Study Steering Committee.

**Table 1:** Participant characteristics.

*N*	100
Sex (% male)	33
Race (% African American)	21
Age at MRI (years)	54.2 ± 4.3
Education (%)	1.5 middle, 24.6 high, 29.2 vocational, 33.8 college, 10.9 post graduate
Smoking (% smoker)	20
Drinking (% drinker)	5
BMI (kg/m^2^)	31.2 ± 6.4
SBP (mm Hg)	119.7 ± 14.5
DBP (mm Hg)	76.0 ± 8.6
Hemoglobin A1c (%)	5.7 ± 1.1
Fasting glucose level (mg/dL)	96.0 ± 21.6
HOMA-IR score	3.5 ± 2.9
Fasting insulin level (mIU/mL)	13.6 ± 9.7
APOE-ε4 (% APOE-ε4/ε4 or ε4/ε3 carriers)	26
Gray matter volume on MRI (% of TCV)	43.3 ± 1.62
White matter volume on MRI (% of TCV)	36.4 ± 1.49
WMH volume on MRI (% of TCV)	0.04 ± 0.05
Z-standardized mean score for all cognitive measures	1.9 ± 3.7
Digit span forwards score	11.8 ± 2.3
Digit span backwards score	7.9 ± 2.0
Logical memory I total score	22.7 ± 6.3
Logical memory II total score	17.4 ± 6.2
Logical memory II recognition total score	24.6 ± 2.4
Trail Making Test A (seconds)	24.9 ± 7.7
Trail Making Test B (seconds)	56.5 ± 23.7
Digit coding score	64.9 ± 14.9
Vocabulary score	31.0 ± 9.0
Word reading score	43.1 ± 7.8

SBP, systolic blood pressure; DBP, diastolic blood pressure; BMI, body mass index; WMH, white matter hyperintensities; TCV, total cranial volume.

### 2.2. Clinical measurements

Validated questionnaires were used to obtain demographic and lifestyle variables, specifically, age, race, sex, cigarette smoking, and alcohol consumption. Adiposity was characterized by the calculation of Body Mass Index, BMI (kg/m^2^) from the height and weight collected by a stadiometer. Duplicate measures of height and weight for each study participant were used to calculate BMI. Similarly, the calculated arithmetic average of blood pressure triplicate measures obtained on the right arm of the participants in a relaxed, sitting position using sphygmomanometers was used to calculate systolic and diastolic blood pressure (SBP and DBP). APOE genotyping was performed directly in the collected serum sample from venipuncture using a method based on isoelectric focusing of delipidated serum followed by immunoblotting using rabbit antihuman APOE antiserum (Srinivasan et al., 2001) Fasting measures of hemoglobin A1c, fasting glucose, HOMA-IR, and fasting insulin were collected using standardized methods (Foster and Berenson, 1987).

### 2.3. Cognitive measurements

Cognitive tests included logical memory I (narrative memory free recall), logical memory II (long term narrative memory free recall), and logical memory II R (long term memory recognition) from the Wechsler Memory Scale III; digit span forward and backward from the Wechsler Adult Intelligence Scale III as well as Trail Making Tests A and B. A global cognition composite score was calculated by averaging the z-scores of each of the domain tests (Lynall et al., 2010; Mayer et al., 2011). Lesser scores on all cognitive measures except the Trails Making Tests are indicators of poorer cognitive health.

### 2.4. Structural MRI acquisition and processing

Brain MRI scans were performed on a GE Discovery 3 T scanner at Pennington Biomedical Research Center. T1-weighted structural MPRAGE (voxel size, 1 × 1 × 1 mm^3^; voxel array, 256 × 256 × 176; flip angle, 8 degrees; NEX, 1) and 2D FLAIR (voxel size, 0.9 × 0.9 × 3 mm^3^; voxel array, 256 × 256 × 50; flip angle, 111 degrees; NEX, 1) images were acquired and analyzed using in-house software, which has been described elsewhere (Yoshita et al., 2006; DeCarli et al., 2008; Carmichael et al., 2012, 2019). Key FLAIR processing steps include manual removal of non-brain elements from the FLAIR image by operator guided tracing of the dura mater within the cranial vault, resulting in delineation of a total cranial volume (TCV) region; MRI non-uniformity correction of the TCV (DeCarli et al., 1996); thresholding of TCV into brain and non-brain tissues (DeCarli et al., 1992); fitting a single Gaussian distribution to the brain tissue intensity distribution and labeling of all voxels with intensity >3.5 standard deviations above the mean as white matter hyperintensities (WMH; DeCarli et al., 2005). Key T1-weighted image processing steps include MRI non-uniformity correction (Fletcher et al., 2012a); and segmentation of gray matter (GM), white matter (WM), and cerebrospinal fluid (CSF) by a Bayesian maximum-likelihood expectation–maximization algorithm (Fletcher et al., 2012b). The primary measures of interest in subsequent analysis were volumes of WMH, GM, and WM, each expressed as a percentage of TCV.

### 2.5. Functional MRI acquisition and preprocessing

Axial 2D gradient echo EPI BOLD were acquired for both task-based and resting-state fMRI (voxel size, 3.5 × 3.5 × 3.5 mm^3^; voxel array, 64 × 64 × 44; flip angle, 90 degrees; TE, 30 ms; TR, 3000 ms; NEX, 1). Two hundred and 160 volumes were acquired over the course of task execution and rest, respectively. Preprocessing of fMRI included slice timing correction, head motion correction (head rotation was required to be <1.5 degree and translation was required to be <1.5 mm at every fMRI time point. All fMRI data sets in this study met that criterion.), smoothing, co-registration to the T1-weighted image, and warping of T1-weighted data to a standard coordinate frame (using Statistical Parametric Mapping 12). Cardiac and respiratory time series were regressed out of the data using RETROICOR and REST Toolkit (Glover et al., 2000; Song et al., 2011). Twenty four regions of interest (ROIs) identified in previous fMRI studies as involved in execution of the Stroop task (Sheu et al., 2012) and 33 ROIs previously identified as default mode network (DMN) regions in resting-state fMRI studies (Buckner et al., 2008; Andrews-Hanna et al., 2010; Alves et al., 2019) were identified, and a single summary fMRI time series was extracted from each ROI using a 3 × 3 × 3 block of voxels in each scan by in-house MATLAB script for EC and FC analysis.

### 2.6. Stroop task

The Stroop task tested inhibitory control in the context of negative feedback and time-pressured responses (Sheu et al., 2012). In each trial, for 400–5,000 ms participants saw one probe word and four target words that were names of colors. The task was to identify the target word whose color matched that of the probe. In the congruent (incongruent) condition, word meaning matched (did not match) the color it was printed in. Correct (incorrect) responses on 3 consecutive incongruent trials prompted a 300 ms reduction (increase) in stimulus duration. Four 52–60 s incongruent trial blocks were interleaved with 4 congruent trial blocks, each of which had the same number of trials as the previous incongruent block. The inter-block interval was 10–17 s.

### 2.7. Resting-state fMRI

Resting-state fMRI was collected for 8 min using the same pulse sequence parameters as the task-based fMRI data. Participants were instructed to keep their eyes open and to stare at a white crosshair on a black background throughout acquisition.

### 2.8. Connectivity analysis

#### 2.8.1. Effective connectivity

The deep stacking network method used to estimate nonlinear Granger causality from the fMRI time series at a source region to that of a target region conditioned on the time series at other source regions has been described previously (Chuang et al., 2021, 2022). The code that supported the findings of this study are available from the corresponding author upon reasonable request. Briefly, the Granger causality of source ROI1 to target ROI2, conditioned on other sources ROIs (ROI1→ROI2|ROIs), is defined in terms of the reduction in prediction error when ROI1, ROI2, and other sources ROIs are used to reconstruct ROI2, compared to prediction error when only ROI2 and other sources ROIs are used to reconstruct ROI2. If incorporating ROI1 improves the reconstruction of ROI2 after accounting for effects of ROI2 and other sources ROIs, the Granger causality index $GC_{index}{}_{ROI1\to ROI2|ROIs}$ will be a larger positive number. Complex causal relationships among several time series can be disentangled by calculating conditional Granger causality with differing assignments of time series to the roles of ROI1, ROI2, and other sources ROIs. To reconstruct target time series from source time series, we used deep stacking networks, which consist of a set of convolutional neural network modules, each trained to reconstruct one time series based on another. Given time series from source and target regions collected from all participant fMRI scans, we used K-fold cross validation to train the deep stacking network K times, each time quantifying $GC_{index}{}_{ROI1\to ROI2|ROIs}\;$within the test data. We consider the evidence for a particular conditional Granger causal relationship strong at the group level when the mean of these $GC_{index}{}_{ROI1\to ROI2|ROIs}$ estimates is statistically significantly >0 in a one-tailed student’s *t*-test (value of *p* < 0.05). Each such causal relationship resulted in an edge originating at the ROI1 node, terminating at the ROI2 node, in the group level graph. The group level graph was constructed for the purpose of visualizing overall trends in causal relationships across the entire set of scans. We also calculated individual-level graphs that allowed us to quantify graph metrics from each scan. To construct an individual-level graph we started by omitting the individual’s scan from the overall data set and randomly partitioning the remainder into K disjoint sets. The deep stacking network was trained on each of the K disjoint sets, and $GC_{index}{}_{ROI1\to ROI2|ROIs}\;$was quantified from just the omitted individual’s scan. We consider the evidence for a particular conditional Granger causal relationship strong when the mean of the K $GC_{index}{}_{ROI1\to ROI2|ROIs}$ estimates is statistically significantly >0 in a one-tailed student’s *t*-test (value of *p* < 0.05). Each such significant $GC_{index}{}_{ROI1\to ROI2|ROIs}$ resulted in a directed edge originating from the graph node corresponding to ROI1, terminating at the graph node corresponding to ROI2, in the individual directed graph representing EC relationships.

Inspired by Jia et al. (2016) and Zamora Esquivel et al. (2019), we used CNN-ACKs in our DSNs architecture to estimate causal relationships. An CNN-ACK is trained to transform the source (s) time series into the target time series. An ACK is defined by a dynamic filter that changes its weights automatically depending on the data in the source time series. The ACK is generated by convolving filters with source time series and using an activation function to transform the result into target time series. The first step is that at each timestep (t), the (1 × 6) hidden layer output is calculated as the dot product of six 1 × 2 filter with the source time series. Then, the Parametric Rectified Linear Unit (PReLU) activation function is applied to each element of hidden layer output to generate the ACK. The estimate of the target time series is the dot product of ACK with the source time series. In each CNN-ACK, the six convolving filters (2 weights and 1 bias terms for each filter) and the parameters of PReLU (6 weights for each timestep) are the learnable parameters. The outputs of each CNN-ACK are provided as inputs to an element-wise weighted sum to produce the final estimate of the target time series. We used the TensorFlow and Keras software packages to build our network architecture and optimized it with the Adam optimizer (*β*1 = 0.9, *β*2 = 0.999) with a learning rate of 0.001 to minimize the loss function of mean squared error between the predicted target time series and the actual target time series (Chollet, 2015; Abadi et al., 2016).

#### 2.8.2. Functional connectivity

We used the Brain Connectivity Toolbox (Rubinov and Sporns, 2010) to calculate conditional FC between a pair of regions, *ROI1* and *ROI2*, while accounting for the effects of all other regions ROIs (*ROI*1 *↔ ROI*2|*ROIs*). Following common practice, the matrix of partial Pearson correlations (Pearson’s r) among all possible *ROI1* and *ROI2* was calculated, and after statistically significant correlation values (value of *p* < 0.05) were retained to construct the individual-level graph (Brier et al., 2014; Meng et al., 2018; Carnevale et al., 2020; Contreras et al., 2020; Zhang et al., 2021). Each such significant conditional FC *ROI*1 *↔ ROI*2|*ROIs* resulted in an undirected link between ROI1 and ROI2 in the graph representing FC relationships. Group consensus graph was calculated as the mean of all individual-level graphs.

### 2.9. Graph metrics

**Figure 1:**
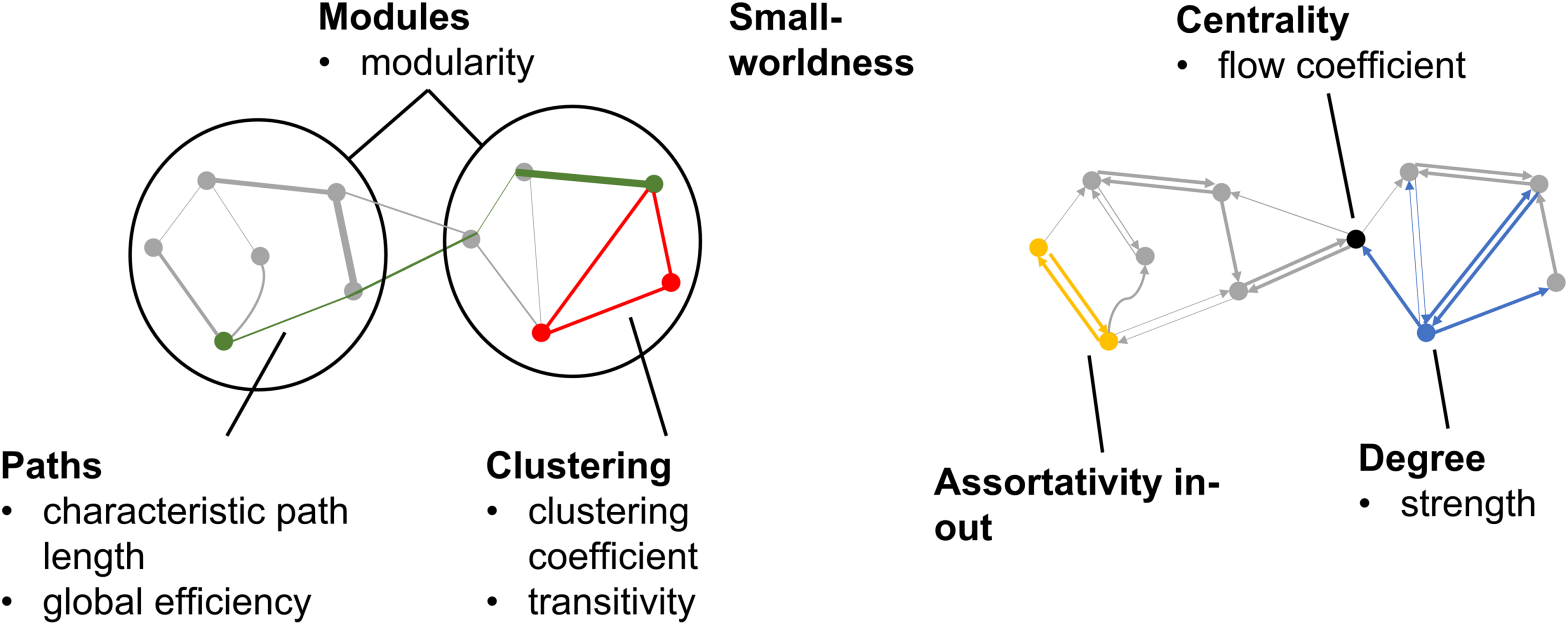
Graph metrics calculated from EC and FC graphs. Categories of graph metrics are shown in bold. Individual graph metrics within each category are listed.

The common global measures graph metrics representing the different aspects of a brain network (Figure 1) were calculated from directed graphs resulting from EC as well as undirected graphs resulting from FC, using the Brain Connectivity Toolbox (Rubinov and Sporns, 2010). The definition of each graph metric can be found in Appendix Table A1. Each graph metric had analogs for both directed and undirected graphs. In previous brain networks studies (Achard and Bullmore, 2007; Meunier et al., 2009; Sanz-Arigita et al., 2010; Petti et al., 2013; Brier et al., 2014; Parhizi et al., 2018; Sun et al., 2020), greater degree, clustering coefficient, transitivity, modularity, global efficiency, assortativity in-out, and small-worldness; as well as lower strength, characteristic path length, and flow coefficient; have been associated with better brain health.

### 2.10. Statistical analysis

Statistical analysis focused on relating demographic, lifestyle, cardiometabolic and cognitive measures as predictors to EC and FC based graph metrics as outcomes. The associations between predictors and outcomes were evaluated in multivariable linear regression models, and statistically significant associations at the value of *p* < 0.05 level are reported in the results. Each model included gender, race, and age at the time of MRI as nuisance covariates, along with one cardiometabolic or cognitive predictor. The set of cardiometabolic and cognitive predictors included BMI, SBP, DBP, APOE-ε4, hemoglobin A1c, fasting glucose, HOMA-IR, fasting insulin, volumes of WMH, GM, and WM, z-standardized mean score for all cognitive measures, digit span forwards/backwards score, logical memory I/II/II recognition total score, digit coding score, vocabulary score, word reading score. We evaluated differences between analogous EC and FC metrics qualitatively, in terms of differences in how they related to the demographic, cardiometabolic and cognitive predictors.

## 3. Results

**Figure 2:**
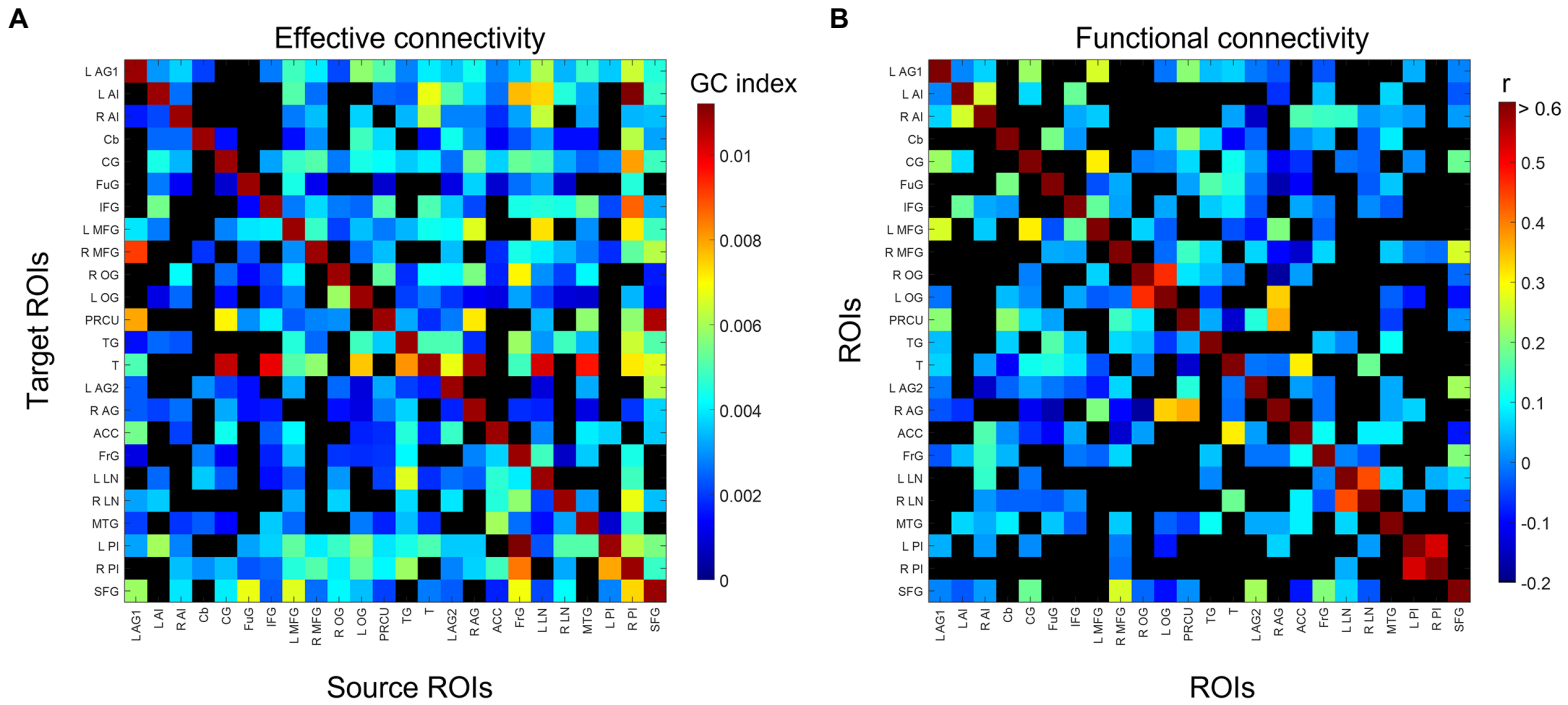
Identified group-consensus **(A)** EC (directed graph) and **(B)** FC (undirected graph) among 24 task-related ROIs for Stroop task fMRI. The connectivity is shown in black if it is not statistically significant. AG, angular gyrus; AI, anterior insula; Cb, cerebellum; CG, cingulate gyrus; FG, fusiform gyrus; IFG, inferior frontal gyrus; MTG, middle frontal gyrus; OG, occipital gyrus; P, precuneus; TG, temporal gyrus; T, thalamus; ACC, anterior cingulate cortex; FG, frontal gyrus; LN, lentiform nucleus; MTG, middle temporal gyrus; PI, posterior insula; SFG, superior frontal gyrus.

**Figure 3:**
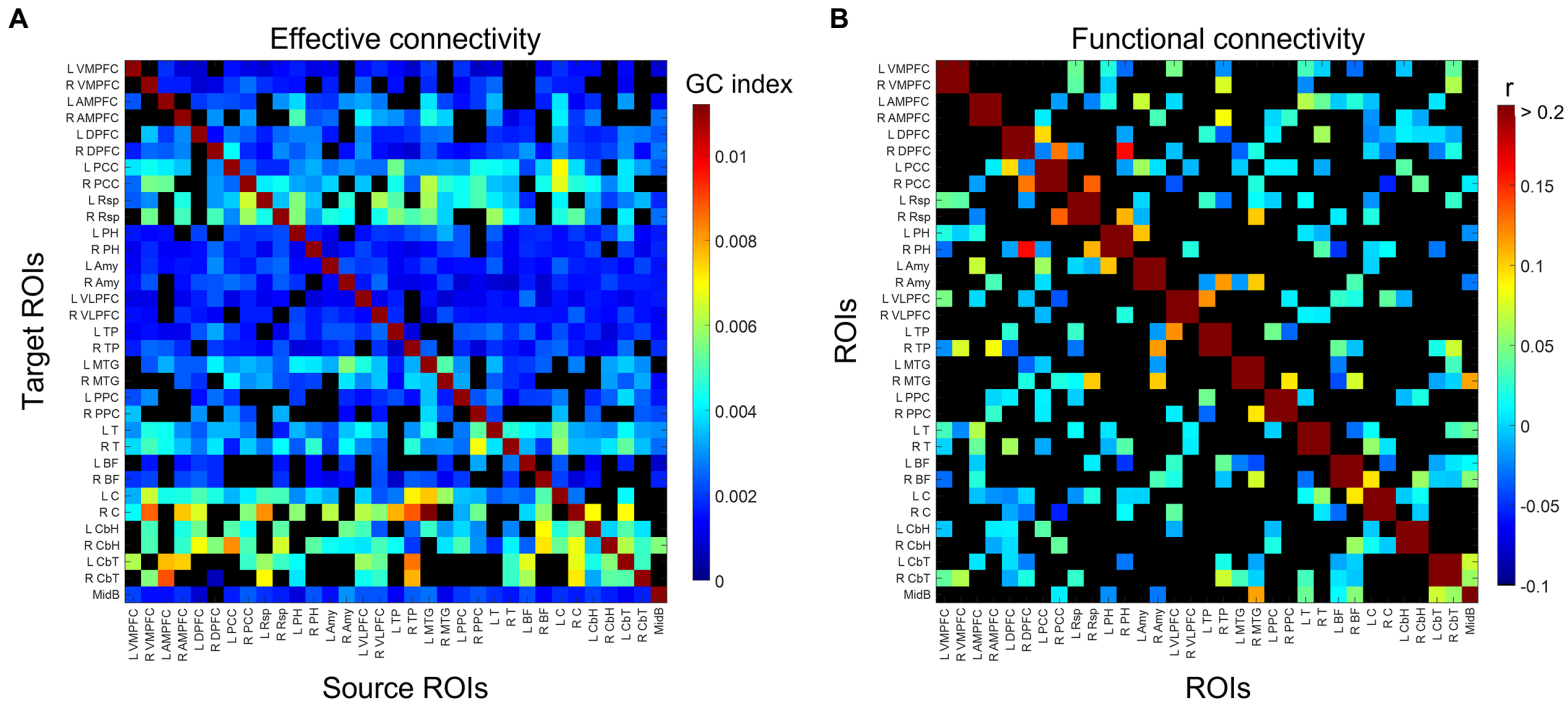
Identified group-consensus **(A)** EC (directed graph) and **(B)** FC (undirected graph) among 33 DMN ROIs for resting-state fMRI. The connectivity is shown in black if it is not statistically significant. VMPFC, ventro-median prefrontal cortex; AMPFC, antero-median prefrontal cortex; DPFC, dorsal prefrontal cortex; PCC, posterior cingulate cortex; Rsp, retrosplenial cortex; PH, parahippocampal region; Amy, amygdala; VLPFC, ventrolateral prefrontal cortex; TP, temporal pole; MTG, middle temporal gyrus; PPC, posterior parietal cortex; T, thalamus; BF, basal forebrain; C, caudate; CbH, cerebellar hemisphere; CbT, cerebellar tonsil; MidB, midbrain.

The group-consensus EC and FC graphs for Stroop task are shown in Figure 2. Figure 3 shows the group-consensus EC and FC graphs for resting-state fMRI. For both Stroop task and resting-state fMRI, almost all of the edges in the group-consensus EC graph were significant; however, few of the edges in the group-consensus FC graph were significant. A specific pattern of several ROIs appearing to be a source for specific target ROIs, including left and right caudate, cerebellar hemisphere, and cerebellar tonsil, has been identified in group-consensus EC for resting-state fMRI. No specific pattern has been found for the rest of the group-consensus graphs. The means and standard deviations of graph metric over all participants have been shown in Table 2. The associations between demographic, cardiometabolic, and cognitive measures and EC/FC based metrics for Stroop task fMRI are shown in Table 3. The associations between demographic, cardiometabolic, and cognitive measures and EC/FC based metrics for resting-state fMRI are shown in Table 4. Figure A1 and Table A2 show the associations between demographic, cardiometabolic, and cognitive measures and EC/FC based metrics among 24 task-related ROIs for Stroop task fMRI and eight core DMN ROIs for Stroop task fMRI.

**Table 2:** The means and standard deviations of graph metric for all participants.

	Degree	Clustering coefficient	Modularity	Transitivity	Global efficiency	Assortativity in-out	Small-worldness	Strength	Characteristic path length	Flow coefficient
EC for Stroop task fMRI	285.4 ± 21.6	0.59 ± 0.03	0.13 ± 0.02	0.59 ± 0.03	0.74 ± 0.02	0.04 ± 0.04	1.05 ± 0.12	13.7 ± 4.9	1.47 ± 0.05	0.18 ± 0.01
FC for Stroop task fMRI	112.2 ± 10.6	0.67 ± 0.03	0.21 ± 0.03	0.64 ± 0.02	0.69 ± 0.03	0.06 ± 0.07	1.06 ± 0.13	21.6 ± 1.1	1.54 ± 0.05	0.44 ± 0.03
EC for resting-state fMRI	533.0 ± 40.9	0.57 ± 0.04	0.11 ± 0.01	0.57 ± 0.04	0.74 ± 0.02	0.01 ± 0.03	1.03 ± 0.02	16.3 ± 6.2	1.48 ± 0.04	0.19 ± 0.01
FC for resting-state fMRI	132.9 ± 18.5	0.64 ± 0.03	0.30 ± 0.05	0.56 ± 0.02	0.60 ± 0.03	0.12 ± 0.08	1.23 ± 0.11	29.6 ± 1.7	1.85 ± 0.10	0.52 ± 0.02

EC, effective connectivity; FC, functional connectivity.

**Table 3:** Linear relationships between demographic, cardiometabolic, and cognitive measures and graph metrics derived from EC and FC for Stroop task fMRI.

	Degree	Clustering coefficient	Modularity	Transitivity	Global efficiency	Assortativity in-out	Small-worldness	Strength	Characteristic path length	Flow coefficient
Gender (*M* = 0, *F* = 1)						EC (*β* = 0.016, *p* = 0.043)				FC (*β* = −0.014, *p* = 0.047)
Race (WA = 0, AA = 1)						EC (*β* = −0.022, *p* = 0.022)		EC (*β* = 2.768, *p* = 0.042)		
SBP			EC (*β* = 0.001, *p* = 0.015)				EC (*β* = 0.001, *p* = 0.018)		EC (*β* = 0.001, *p* = 0.038)	
DBP	EC (*β* = −0.877, *p* = 0.001)	EC (*β* = −0.001, *p* = 0.005)	EC (*β* = 0.001, *p* = 0.008)	EC (*β* = −0.001, *p* = 0.003)	EC (*β* = −0.001, *p* = 0.002)				EC (*β* = 0.002, *p* = 0.001)	
APOE-ε4							FC (*β* = −0.046, *p* = 0.026)	FC (*β* = 0.600, *p* = 0.032)		
Hemoglobin A1c								FC (*β* = −0.223, *p* = 0.029)		
WMH volume			FC (*β* = −26.3, *p* = 0.001)					EC (*β* = 2,172, *p* = 0.021)	FC (*β* = −31.6, *p* = 0.017)	
Digit span backwards							FC (*β* = −0.009, *p* = 0.041)			FC (*β* = 0.003, *p* = 0.043)
Vocabulary		EC (*β* = −0.001, *p* = 0.017)		EC (*β* = −0.001, *p* = 0.025)						

EC, effective connectivity; FC, functional connectivity; M, male; F, female; WA, white American; AA, African American; SBP, systolic blood pressure; DBP, diastolic blood pressure; WHM, white matter hyperintensities.

**Table 4:** Linear relationships between demographic, cardiometabolic, and cognitive measures and graph metrics derived from EC and FC for resting-state fMRI.

	Degree	Clustering coefficient	Modularity	Transitivity	Global efficiency	Assortativity in-out	Small-worldness	Strength	Characteristic path length
Age	EC (*β* = −2.556, *p* = 0.012)	EC (*β* = −0.002, *p* = 0.016)	EC (*β* = 0.001, *p* = 0.038)	EC (*β* = −0.002, *p* = 0.013)	EC (*β* = −0.001, *p* = 0.011)				EC (*β* = 0.002, *p* = 0.010)
Gender (*M* = 0, *F* = 1)						EC (*β* = −0.017, *p* = 0.017)		FC (*β* = −1.145, *p* = 0.004)	
Alcohol (non-drinker = 0, drinker = 1)							EC (*β* = −0.036, *p* = 0.015)	FC (*β* = 2.522, *p* = 0.009)	
BMI								EC (*β* = 4.167, *p* = 0.015)	
Fasting glucose								EC (*β* = 3.475, *p* = 0.035)	
HOMA-IR								EC (*β* = 7.011, *p* = 0.003)	
Fasting insulin								EC (*β* = 7.033, *p* = 0.004)	
WMHs volume								EC (*β* = 3.125, *p* = 0.025)	
Logical memory II total score								EC (*β* = 3.416, *p* = 0.044)	
Word reading								EC (*β* = 3.446, *p* = 0.038)	

EC, effective connectivity; FC, functional connectivity; M: male; F, female; WHM, white matter hyperintensities. Neither EC or FC based small-worldness and flow coefficient associated with demographic, cardiometabolic and cognitive risk factors.

### 3.1. Associations with demographic measures

#### 3.1.1. Stroop task fMRI

Age at MRI was not associated with either EC based metric (minimum value of *p* = 0.059), or FC based metric (minimum value of *p* = 0.065). Better EC based assortativity in-out (value of *p* = 0.043) and better FC based flow coefficient (value of *p* = 0.047) were found for females compared to males. Better EC based assortativity in-out and strength (value of *p* = 0.022 and 0.042, respectively) were found for white Americans compared to African Americans.

#### 3.1.2. Resting-state fMRI

Age at MRI was associated with EC based metrics but not with any FC based metric. Greater age was associated with worse EC based degree, clustering coefficient, transitivity, global efficiency, and characteristic path length (maximum value of *p* = 0.038). Gender was associated with both EC and FC based metrics. Better EC based assortativity in-out (value of *p* = 0.017) was found for males, but better FC based strength (value of *p* = 0.004) was found for females. Self-reported history of drinking was associated with both EC and FC based metrics. Better EC based small-worldness (value of *p* = 0.015) and better FC based strength (value of *p* = 0.009) was found for non-drinkers. Better EC based assortativity in-out (*p-*value = 0.017) was found for males, but better FC based strength (*p-*value = 0.004) was found for females. Race, education, and smoking history were not associated with any EC based metric (minimum *p-*value = 0.068), nor any FC based metric (minimum *p-*value = 0.077).

### 3.2. Associations with cardiometabolic measures

#### 3.2.1. Stroop task fMRI

Systolic and diastolic blood pressure were associated with EC based metrics but not with any FC based metric. Greater SBP was associated with worse EC based characteristic path length (*p*-value = 0.038), but with better EC based modularity and small-worldness (maximum *p*-value = 0.018). Greater DBP was associated with worse EC based degree, clustering coefficient, transitivity, global efficiency, and characteristic path length (maximum *p*-value = 0.008). Instead, APOE-ε4 was not associated with any EC based metric, but with FC based metrics. Worse FC based small-worldness and strength were found in ε4 allele carriers (*p*-value = 0.026 and 0.032, respectively). Greater hemoglobin A1c were associated with worse FC based strength (maximum *p*-value = 0.029). Moreover, white matter hyperintensities volume was associated with both EC and FC based metrics. Greater WMH was associated with worse EC based strength (*p*-value = 0.021), FC based modularity and characteristic path length (*p*-value = 0.001 and 0.017, respectively). BMI, glycemic measures, and gray/white matter volume were not associated with any EC based metric (minimum *p*-value = 0.100), nor any FC based metric (minimum *p*-value = 0.115).

#### 3.2.2. Resting-state fMRI

BMI and WMH were associated with EC based metrics but not with any FC based metric. Greater BMI was associated with worse EC based strength (*p*-value = 0.015). Greater fasting glucose, HOMA-IR, and fasting insulin were associated with worse EC based strength (maximum *p*-value = 0.035). Greater WMH was associated with worse EC based strength (*p*-value = 0.025). Systolic and diastolic blood pressure, APOE-ε4, and gray/white matter volume were not associated with any EC based metric (minimum *p*-value = 0.052), nor any FC based metric (minimum *p*-value = 0.137).

### 3.3. Associations with cognitive measures

#### 3.3.1. Stroop task fMRI

Vocabulary score was associated with EC based metrics but not with any FC based metric. Greater vocabulary score was associated with worse EC based clustering coefficient and transitivity (*p*-value = 0.017 and 0.025, respectively). Instead, digit span backwards score was not associated with any EC based metric, but with FC based metrics. Greater digit span backwards score was associated with worse FC based small-worldness and flow coefficient (*p*-value = 0.041 and 0.043, respectively). None the rest of the cognitive measures were associated with any EC based metric (minimum *p*-value = 0.068), nor any FC based metric (minimum *p*-value = 0.097).

#### 3.3.2. Resting-state fMRI

Logical memory II total score and word reading score were associated with EC based metrics but not with any FC based metric. Greater logical memory II total score and word reading score were associated with worse EC based strength (*p*-value = 0.044 and 0.038, respectively). None the rest of the cognitive measures were associated with any EC based metric (minimum *p*-value = 0.250), nor any FC based metric (minimum *p*-value = 0.228).

## 4. Discussion

In a cohort of nominally healthy middle aged individuals, almost all of the edges in the group-consensus EC graph were significant; however, few of the edges in the group-consensus FC graph were significant. We believe this difference is because EC can identify a wider range of significant relationships than FC can: EC can identify causal relationship across a wide range of time lags between the source region and the target region; FC, on the other hand, only accounts for synchrony (i.e., zero time lag) relationships between the regions. Also, EC and FC had differential associations with demographic, cardiometabolic, and cognitive measurements in both task-based and resting-state fMRI. The task-based results suggested that certain health-related measures associated specifically with EC metrics, and others associated specifically with FC metrics. The resting-state results similarly suggested differential associations between EC and FC, and that EC metrics had more associations with health-related measures than FC metrics did. There are several ways in which the imposition of task conditions could affect EC and FC values. First, EC and FC may have a different temporal structure in task fMRI data due to the time-varying nature of task conditions, which lead to time-varying cognitive loads on various brain regions. EC and FC may have different spatial structures as well when applied to task fMRI data, as the task demands may force the brain to recruit different brain regions for execution. Finally, the imposition of task conditions could cause greater fluctuations in the BOLD signal than are seen during rest, and this amplitude difference may by itself cause differences between task and rest connectivity measures. Therefore, the first implication of these findings is that future studies of midlife brain health should consider both EC and FC analyses to get a more complete picture of functional network related aspects of brain health. The second implication is that future studies of midlife brain health should consider collecting both task-based and resting-state fMRI scans, again to get a more complete picture of relevant aspects of brain health.

Our results shows that either EC or FC showed a significant correlation with the network metric but not both ─ there was no case where both FC and EC showing a significant correlation. Differential associations for EC compared to FC are plausible, given that they are quantifying distinct properties of the underlying fMRI signals. A key difference between EC and FC analysis is that EC analysis can assess a specific form of causal relationships, while FC analysis captures correlation (Stigler, 2005; Altman and Krzywinski, 2015; Moreau and Dumas, 2021). Altman et al. has pointed out that a causal relationship (EC) can arise between variables in the presence or absence of a correlation (FC), and therefore we cannot equate causality with correlation in either direction (Altman and Krzywinski, 2015). This key difference, in theory, could account for more statistically significant edges and differential associations for EC compared to FC. Our results suggest that this difference between what EC and FC calculates is actually relevant to real-world data sets containing fMRI and health information. Thus, we suggest calculating EC as complementary to FC analyses. Calculating both types of metrics adds nothing to acquisition time but does add to the computational burden of post-processing.

Our findings of relationships between demographic measures (age and gender) and EC or FC aligned well with previous literature. In general, greater degree, clustering coefficient, transitivity, modularity, global efficiency, assortativity in-out, and small-worldness; as well as lower strength, characteristic path length, and flow coefficient; have been associated with better brain health (Achard and Bullmore, 2007; Meunier et al., 2009; Sanz-Arigita et al., 2010; Petti et al., 2013; Brier et al., 2014; Parhizi et al., 2018; Sun et al., 2020). Greater degree suggests a larger number of functional connections between the current region and other regions. Greater clustering coefficient, transitivity, modularity, and lower flow coefficient suggest greater inter-regional connectivity among a set of regions. Greater modularity, global efficiency, and small-worldness, as well as lower characteristic path length, suggest greater efficiency of functional network organization from the perspective of information transfer across the network. Greater assortativity in-out coefficient suggests that the brain regions tend to connect to other brain regions that have similar degree. Lower strength suggests that a region has several weak functional connections with a large set of other regions, rather than a few strong connections. Prior studies demonstrated lower FC based degree and modularity, and lower EC based small-worldness, in middle-aged or old research participants compared to young participants, based on resting-state fMRI (Meunier et al., 2009; Petti et al., 2013; Song et al., 2014; Archer et al., 2016). Our results similarly suggested that EC based metrics were intuitively associated with age within this cohort of middle aged individuals, based on resting-state scans. Young age is usually correlated with better brain health in healthy populations. Also, resting-state FC studies have reported that women may have greater graph node degree within the default mode network, compared to men (Bluhm et al., 2008; Allen et al., 2011; Zhang et al., 2018); we similarly found better EC and FC based metrics among women, compared to men, in both task-based and resting-state scans. To our knowledge, there have been no reported observations of race differences in EC or FC to date in middle aged individuals; we report what may be the first finding of poorer task-based EC among African Americans compared to corresponding whites.

Many of our results for cardiometabolic measures (blood pressure, BMI, WMH, APOE-ε4) aligned well with previous literature. We report a lack of association between FC and blood pressure measures, similar to prior studies showing no association to blood pressure (Song et al., 2011; Zhou et al., 2011; Hinault et al., 2019) as well as others showing no differences between hypertensive and normotensive groups (Carnevale et al., 2020). We also report significant associations between blood pressure and EC, as in prior studies (Chand et al., 2017; Bu et al., 2018). Our finding of an association between worse resting-state EC and greater BMI is reminiscent of an earlier finding of worse resting-state EC among obese young adults compared to normal-weight young adults (Duan et al., 2020), although we did not replicate earlier findings of reduced resting-state FC among obese young adults (Baek et al., 2017; Meng et al., 2018; Ottino-González et al., 2021). We report that greater WMH burden is associated with poorer EC during task and rest along with poorer FC during rest. We believe that methodological differences between studies may account for many of these discrepancies. For example, some prior studies (Chen et al., 2019, 2021) focused solely on FC between the thalamus and the whole brain, while other studies explored FC solely within the default mode network. Standardizing fMRI post-processing pipelines to minimize such methodological differences has been notoriously difficult. We are providing what may be one of the first reports of significant associations between APOE-ε4 carrier status and task-based FC. APOE carrier status has previously been shown to be associated with a variety of different indicators of poorer neurobiological health, including degradation of synaptic and neuronal function (Contreras et al., 2020; Turney et al., 2020). Worse FC-task based graph metrics were found in ε4 allele carriers, suggesting that such APOE-related decrements in neuronal and synaptic health may culminate in connectivity deficits. Overall, our results align well with the intuitive notion that better graph metrics should associate with indicators of better brain health. These results have substantial agreement with prior literature, thus lending some plausibility to the current findings. Discrepancies between our findings and previous reports could be accounted for by numerous methodological and study population differences. Moreover, several prior studies reported the associations between race and APOE-e4 (Beydoun et al., 2021; Weiss et al., 2021). However, there was no statistically significant correlation between the graph metrics and the interaction terms of race and APOE-e4. Prior studies that suggest such interactions generally assessed cognitive outcome measures while our outcomes are brain connectivity variables; we speculate that interactive effects on cognition may be exerted through other mechanisms besides brain connectivity.

Our finding of a significant association between one type of cognitive measures (digit span backwards/forwards scores) and task-based FC aligns well with prior reports with task-based fMRI data (Ginestet and Simmons, 2011; Stanley et al., 2015). We are unaware of any prior reports on associations between EC and cognitive function, and provide what may be one of the first reports of such associations here. The closest we can get to this finding in the current literature is the literature on FC in disease populations, including mild cognitive impairment and Alzheimer’s disease (Grady et al., 2003; Wang et al., 2006; Zhang et al., 2017). Several of these studies reported that FC is actually greater in those with worse disease status, suggesting that elevating FC may be a compensatory mechanism triggered by the disease state. Similarly, we found that better cognitive function scores were associated with worse EC-based graph metrics. While there have been numerous reports of associations between resting-state FC and cognitive functioning or differences in resting-state FC between cognitively healthy and unhealthy groups (Van Den Heuvel and Pol, 2010; Liang et al., 2011; Zhou et al., 2013; Zhang et al., 2017), these prior studies largely did not take place entirely within a cognitively healthy middle-aged population. This difference from prior literature may account for our lack of finding of such associations in our data.

A key strength of the study is its comprehensive nature, with comparisons among multiple forms of brain connectivity (EC and FC), both task-based and resting-state fMRI, and multi-faceted assessment of individuals in terms of demographics, cardiometabolic risks, and cognition. The use of an established, deeply characterized population-based cohort is another key strength. Future studies should consider longitudinal measurement of cardiovascular and cognitive measures from as young an age as possible. One limitation to this study is the relatively small sample size of the dataset (100 participants). It would be helpful to verify the robustness of the results with a public dataset with larger sample size. Another limitation is our ROI-based approach to calculating EC and FC, i.e., we only calculated connectivity among regions previously identified as activated by the Stroop task, or among those previously identified as being in the default mode network. This approach may miss certain interesting functional connections outside of the known task-related regions, but unlike whole-brain analyses it offers a lower risk of the false positives that have contributed to the replication crisis currently roiling the fMRI field (Bennett and Miller, 2010; Sheu et al., 2012). The other possible limitation is that we did not adjust for inter-individual or inter-regional differences in the hemodynamic response function to stimuli. Some studies have suggested that such adjustments are important (Smith et al., 2012; Rangaprakash et al., 2017), while others suggest they are irrelevant (Seth et al., 2013; Wen et al., 2013).

## 5. Conclusion

In a diverse, cognitively healthy, middle-aged community sample, graph metrics derived from EC based directed graphs and FC based undirected graphs in both task-based and resting-state scans associated differentially with recognized demographic, cardiometabolic and cognitive indicators of brain health.

## Data availability statement

The raw data supporting the conclusions of this article will be made available by the authors, without undue reservation.

## Ethics statement

The studies involving human participants were reviewed and approved by Pennington Biomedical Research Center. The patients/participants provided their written informed consent to participate in this study.

## Author contributions

K-CC and OC: experimental design, data interpretation, and manuscript drafting. K-CC, SR, KaM, JS, KeM, KG, RD, and LB: data collection. K-CC: data analysis. All authors contributed to the article and approved the submitted version.

## Funding

Funding for this work was supported by National Institutes of Health grants R01AG041200 and R01AG062309 as well as the Pennington Biomedical Research Foundation, Baton Rouge, LA.

## Conflict of interest

The authors declare that the research was conducted in the absence of any commercial or financial relationships that could be construed as a potential conflict of interest.

## Publisher’s note

All claims expressed in this article are solely those of the authors and do not necessarily represent those of their affiliated organizations, or those of the publisher, the editors and the reviewers. Any product that may be evaluated in this article, or claim that may be made by its manufacturer, is not guaranteed or endorsed by the publisher.
