# Supplementary material for: Brain effective connectivity and functional connectivity as markers of lifespan vascular exposures in middle-aged adults: The Bogalusa Heart Study
Source: Front Aging Neurosci. 2023 Mar 14;15:1110434. doi: 10.3389/fnagi.2023.1110434 (PMC10043334; doi:10.3389/fnagi.2023.1110434)
Supplement: Supplementary file 1 [file Data_Sheet_1.docx]

**Appendix**

**Table A1** The definitions of graph metrics (Rubinov and Sporns, 2010).

| Metric | Directed graph | Undirected graph |
| --- | --- | --- |
| Basic concepts and notation | $N$ is the set of all nodes in the network, and $n$ is the number of nodes.  $L$ is the set of all links in the network, and $l$ is number of links.  $a_{ij}$ is a directed link from nodes $i$ to $j$, and weights $w_{ij}$ are GC indices from nodes $i$ to $j, (i,j)\in N.$ | $a_{ij}$ is an undirected link between nodes $i$ and $j$, and weights $w_{ij}$ are partial Pearson’s r between nodes $i$ and $j, (i,j)\in N.$ Please note that $a_{ij}= a_{ji}$ represents an undirected link between nodes $i$ and $j$. |
| Degree | Number of links in the network:  $l=\sum_{i,j\in N} a_{ij}$ | |
| Strength | The sum of weights of links in the network:  $s=\sum_{i,j\in N} w_{ij}$ | |
| Clustering coefficient | Clustering coefficient of the network:  $C=\frac{1}{n}\sum_{i\in N} C_{i}$  where $C_{i}$ is the clustering coefficient of node $i$ (fraction of triangles around a node). | |
| Transitivity | The ratio of triangles to triplets in the network:  $t_{i}=\frac{1}{2}\sum_{j,h\in N} a_{ij}a_{ih}a_{ih}$  $T=\frac{\sum_{i\in N} 2t_{i}}{\sum_{i\in N} l_{i}(l_{i}-1)}$ | |
| Modularity | The extent to which a graph can be divided into clearly separated communities:  $Q=\sum_{u\in M} [e_{uu}-{(\sum_{v\in M} e_{uv})}^{2}]$  where the network is fully subdivided into a set of nonoverlapping modules $M$, and $e_{uv}$ is the proportion of all links that connect nodes in module $u$ with nodes in module $v$. | |
| Characteristic path length | The average shortest path length in the network:  $L=\frac{1}{n}\sum_{i\in N} L_{i}=\frac{1}{n}\sum_{i\in N} \frac{\sum_{j\in N,j\neq i} d_{ij}}{n-1}$  where $d_{ij}$ is the shortest distance between node $i$ and other node $j$. | |
| Global efficiency | The average inverse shortest path length in the network:  $E=\frac{1}{n}\sum_{i\in N} E_{i}=\frac{1}{n}\sum_{i\in N} \frac{\sum_{j\in N,j\neq i} d_{ij}^{-1}}{n-1}$  where $E_{i}$ is the efficiency of node $i$. Global efficiency is a scaled measure ranging from 0–1, with a value of 1 indicating maximum global efficiency in the network. | |
| Flow coefficient | The fraction of all shortest paths in the network that contain a given node. | |
| Assortativity | The correlation coefficient between the degrees of all nodes on two opposite ends of a link. Assortativity is a scaled measure ranging from -1 to 1, with an absolute value of 1 indicating maximum assortativity in the network. | |
| Small-worldness | Network small-worldness:  $S=\frac{C/{C_{rand}}}{L/{L_{rand}}}$  where $C$ and $C_{rand}$ are the clustering coefficients, and $L$ and $L_{rand}$ are the characteristic path lengths of the respective tested network and a random network (the same number of nodes and edges as the tested network). $C_{rand}$ and $L_{rand}$ are the average values over the N random networks. Small-world networks often have $S\gg1$. | |

**
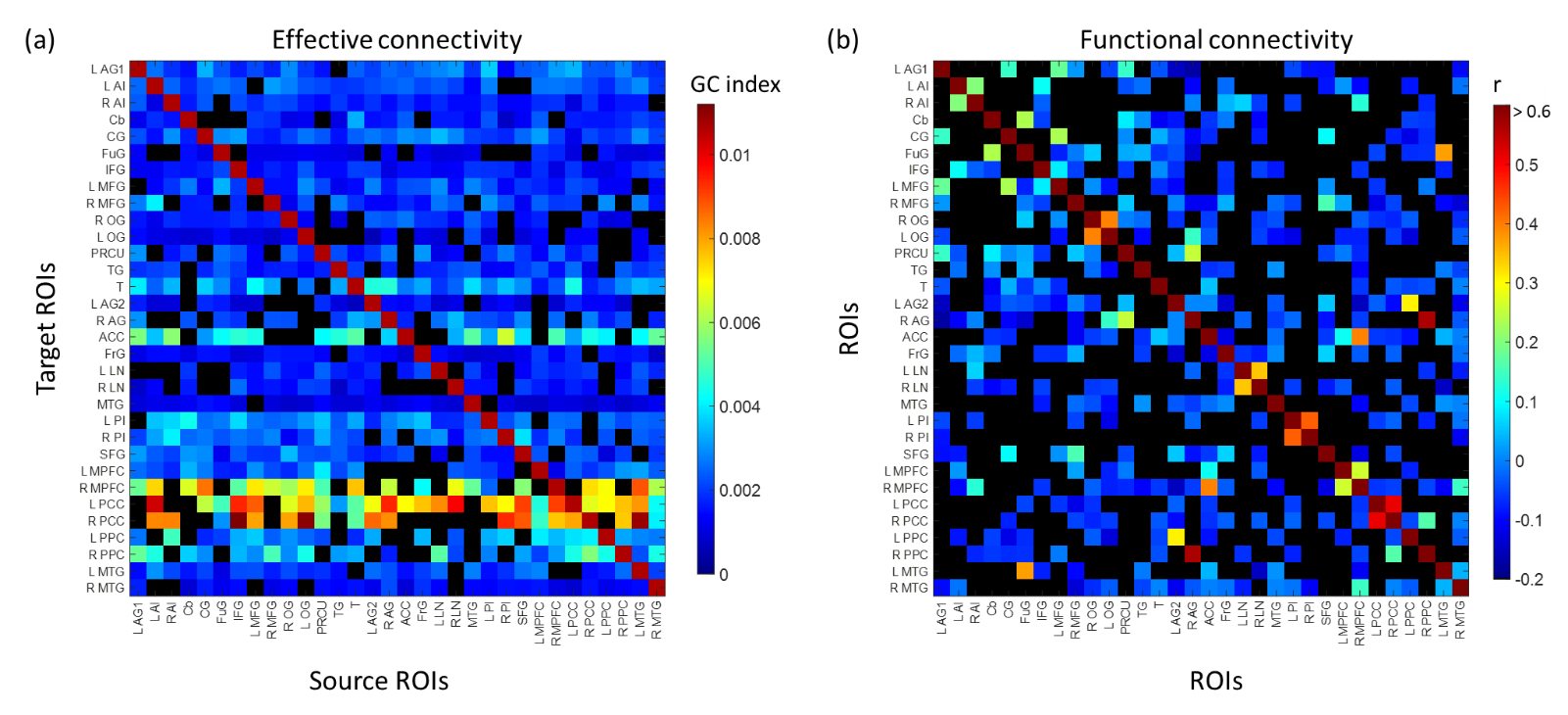
**

**Figure A1** Identified group-consensus (a) EC (directed graph) and (b) FC (undirected graph) among 24 task-related ROIs for Stroop task fMRI and eight core DMN ROIs. The connectivity is shown in black if it is not statistically significant. AG angular gyrus, AI anterior insula, Cb cerebellum, CG cingulate gyrus, FG fusiform gyrus, IFG inferior frontal gyrus, MTG middle frontal gyrus, OG occipital gyrus, P precuneus, TG temporal gyrus, T thalamus, ACC anterior cingulate cortex, FG frontal gyrus, LN lentiform nucleus, MTG middle temporal gyrus, PI posterior insula, SFG superior frontal gyrus, MPFC median prefrontal cortex, PCC posterior cingulate cortex, PPC posterior parietal cortex, MTG middle temporal gyrus.

**Table A2** Linear relationships between demographic, cardiometabolic, and cognitive measures and graph metrics derived from EC and FC among 24 task-related ROIs for Stroop task fMRI and eight core DMN ROIs for Stroop task fMRI.

|  | Degree | Clustering coefficient | Modularity | Transitivity | Global efficiency | Assortativity in-out | Small-worldness | Strength | Characteristic path length | Flow coefficient |
| --- | --- | --- | --- | --- | --- | --- | --- | --- | --- | --- |
| Gender  (M=0, F=1) |  |  |  |  |  | EC  (β=0.002, p=0.043) |  |  |  |  |
| Race  (WA=0, AA=1) |  |  |  |  |  | EC  (β=0.008,  p=0.023) |  | EC (β=3.947,  p=0.049) |  |  |
| SBP |  |  | EC (β=0.001,  p=0.015) |  |  |  | EC (β=0.001,  p=0.020) |  | EC  (β=0.001,  p=0.039) |  |
| DBP | EC  (β=-0.845,  p=0.001) | EC  (β=-0.001,  p=0.006) | EC (β=0.001,  p=0.008) | EC  (β=-0.001,  p=0.003) | EC  (β=-0.001,  p=0.002) |  |  |  | EC  (β=0.001,  p=0.001) |  |
| BMI |  | FC  (β=-0.001,  p=0.035) |  |  |  |  | FC  (β=0.021,  p=0.037) |  |  |  |
| Fasting glucose |  | EC  (β=-0.001,  p=0.043) |  | EC  (β=-0.001,  p=0.046) |  | EC  (β=-0.001,  p=0.041) |  |  |  |  |
| HOMA-IR | EC  (β=-4.976,  p=0.042) | EC  (β=-0.004,  p=0.043) |  |  | EC  (β=-0.003,  p=0.039) |  |  |  | EC  (β=0.005,  p=0.037) |  |
| Fasting insulin |  |  |  |  |  |  | EC  (β=0.001,  p=0.019) |  |  |  |
| White matter volume |  |  |  | FC  (β=0.296,  p=0.030) |  |  |  |  |  | FC  (β=-0.387,  p=0.036) |
| WMH volume |  |  | FC  (β=-19.3,  p=0.027) |  |  |  |  | EC (β=2782,  p=0.045) |  |  |
| Digit span forwards |  |  |  |  |  | EC  (β=0.003,  p=0.013) |  |  |  |  |
| Digit span backwards |  |  |  |  |  |  |  |  |  | EC  (β=-0.001,  p=0.029) |
| Digit coding |  |  |  | FC  (β=0.001,  p=0.026) | FC  (β=0.001,  p=0.046) |  | EC  (β=0.001,  p=0.010) |  |  |  |
| Trail making test A |  |  | FC  (β=-0.070,  p=0.023) |  |  |  |  |  |  |  |
| EC: Effective connectivity, FC: Functional connectivity, M: Male, F: Female, WA: White American, AA: African American, SBP: Systolic blood pressure, DBP: Diastolic blood pressure, WHM: White matter hyperintensities. | | | | | | | | | | |
