# Supplementary material for: Brain effective connectivity and functional connectivity as markers of lifespan vascular exposures in middle-aged adults: The Bogalusa Heart Study
Source: Front Aging Neurosci. 2023 Mar 14;15:1110434. doi: 10.3389/fnagi.2023.1110434 (PMC10043334; doi:10.3389/fnagi.2023.1110434)
Supplement: Supplementary file 2 [file Table_1.DOCX]

**Table S1** Linear relationships between demographic, cardiometabolic, and cognitive measures and graph metrics derived from EC for Stroop task fMRI.

|  | Degree | | Clustering coefficient | | Modularity | | Transitivity | | Global efficiency | | Assortativity in-out | | Small- worldness | | Strength | | Characteristic path length | | Flow coefficient | |
| --- | --- | --- | --- | --- | --- | --- | --- | --- | --- | --- | --- | --- | --- | --- | --- | --- | --- | --- | --- | --- |
|  | p | β | p | β | p | β | p | β | p | β | p | β | p | β | p | β | p | β | p | β |
| Age at MRI | 0.948 | -0.033 | 0.797 | 0.001 | 0.698 | 0.001 | 0.934 | 0.001 | 0.937 | 0.001 | 0.059 | 0.001 | 0.626 | 0.001 | 0.974 | 0.004 | 0.929 | 0.001 | 0.890 | 0.001 |
| Gender  (M=0, F=1) | 0.884 | -0.734 | 0.953 | 0.001 | 0.548 | 0.002 | 0.936 | -0.001 | 0.965 | 0.001 | 0.043 | 0.016 | 0.688 | -0.003 | 0.930 | -0.097 | 0.890 | 0.001 | 0.415 | -0.002 |
| Race  (WA=0, AA=1) | 0.195 | 7.985 | 0.442 | 0.006 | 0.291 | -0.005 | 0.290 | 0.009 | 0.228 | 0.007 | 0.022 | -0.022 | 0.390 | -0.009 | 0.042 | 2.768 | 0.219 | -0.016 | 0.855 | -0.001 |
| Education | 0.272 | -27.49 | 0.211 | -0.045 | 0.640 | 0.009 | 0.209 | -0.045 | 0.335 | -0.024 | 0.831 | 0.008 | 0.935 | -0.012 | 0.789 | -1.261 | 0.334 | 0.050 | 0.844 | 0.002 |
| Smoking (non-smoker = 0, smoker = 1) | 0.709 | 2.825 | 0.691 | 0.004 | 0.704 | -0.002 | 0.843 | 0.002 | 0.700 | 0.003 | 0.360 | 0.010 | 0.792 | 0.012 | 0.354 | 1.129 | 0.692 | -0.006 | 0.623 | 0.002 |
| Alcohol  (non-drinker = 0, drinker = 1) | 0.594 | 7.483 | 0.828 | 0.004 | 0.265 | -0.012 | 0.780 | 0.006 | 0.589 | 0.008 | 0.186 | -0.026 | 0.952 | 0.005 | 0.326 | -2.218 | 0.587 | -0.016 | 0.768 | 0.002 |
| SBP | 0.059 | -0.309 | 0.255 | 0.001 | 0.015 | 0.001 | 0.199 | 0.001 | 0.060 | 0.001 | 0.931 | 0.001 | 0.018 | 0.001 | 0.392 | 0.031 | 0.038 | 0.001 | 0.364 | 0.001 |
| DBP | 0.001 | -0.877 | 0.005 | -0.001 | 0.008 | 0.001 | 0.003 | -0.001 | 0.002 | -0.001 | 0.516 | 0.001 | 0.120 | 0.001 | 0.273 | 0.066 | 0.001 | 0.002 | 0.440 | 0.001 |
| APOE-ε4 | 0.279 | 6.115 | 0.477 | 0.005 | 0.507 | -0.003 | 0.343 | 0.007 | 0.264 | 0.006 | 0.752 | 0.003 | 0.831 | -0.002 | 0.052 | -2.481 | 0.289 | -0.012 | 0.382 | 0.003 |
| BMI | 0.412 | -0.326 | 0.211 | -0.001 | 0.849 | 0.001 | 0.228 | -0.001 | 0.412 | 0.001 | 0.211 | -0.001 | 0.236 | -0.003 | 0.100 | -0.142 | 0.499 | 0.001 | 0.879 | 0.001 |
| Hemoglobin A1c | 0.977 | -0.062 | 0.926 | 0.001 | 0.380 | 0.001 | 0.985 | 0.001 | 0.929 | 0.001 | 0.951 | 0.001 | 0.858 | 0.002 | 0.242 | 0.555 | 0.932 | 0.001 | 0.782 | 0.001 |
| Fasting glucose | 0.998 | 0.001 | 0.954 | 0.001 | 0.285 | 0.001 | 0.943 | 0.001 | 0.848 | 0.001 | 0.851 | 0.001 | 0.266 | -0.001 | 0.525 | 0.015 | 0.962 | 0.001 | 0.551 | 0.001 |
| HOMA-IR | 0.821 | 0.274 | 0.903 | 0.001 | 0.575 | 0.001 | 0.932 | 0.001 | 0.937 | 0.001 | 0.648 | 0.001 | 0.205 | -0.013 | 0.909 | -0.023 | 0.861 | 0.001 | 0.742 | 0.001 |
| Fasting insulin | 0.892 | -0.049 | 0.571 | 0.001 | 0.519 | 0.001 | 0.684 | 0.001 | 0.708 | 0.001 | 0.492 | 0.001 | 0.252 | -0.003 | 0.992 | 0.001 | 0.827 | 0.001 | 0.466 | 0.001 |
| Gray matter volume | 0.736 | 55.49 | 0.753 | 0.060 | 0.618 | -0.064 | 0.756 | 0.072 | 0.767 | 0.049 | 0.892 | 0.036 | 0.761 | 0.082 | 0.571 | -17.23 | 0.842 | -0.069 | 0.818 | -0.018 |
| White matter volume | 0.661 | -73.81 | 0.726 | -0.069 | 0.979 | -0.003 | 0.638 | -0.111 | 0.814 | -0.040 | 0.287 | -0.286 | 0.798 | -0.071 | 0.288 | -32.92 | 0.625 | 0.172 | 0.604 | -0.042 |
| WMH volume | 0.098 | -8881 | 0.261 | -7.070 | 0.486 | 2.963 | 0.120 | -11.75 | 0.054 | -10.41 | 0.391 | 7.381 | 0.335 | 8.455 | 0.021 | 2172 | 0.074 | 20.08 | 0.264 | -2.849 |
| Z-standardized mean score for all cognitive measures | 0.871 | -0.104 | 0.723 | 0.001 | 0.596 | 0.001 | 0.886 | 0.001 | 0.887 | 0.001 | 0.376 | 0.001 | 0.406 | -0.001 | 0.560 | 0.082 | 0.763 | 0.001 | 0.668 | 0.001 |
| Digit span forwards | 0.849 | -0.201 | 0.974 | 0.001 | 0.816 | 0.001 | 0.817 | 0.001 | 0.866 | 0.001 | 0.394 | 0.001 | 0.867 | 0.001 | 0.416 | 0.187 | 0.647 | 0.001 | 0.326 | -0.001 |
| Digit span backwards | 0.131 | -1.797 | 0.296 | -0.002 | 0.184 | 0.001 | 0.213 | -0.002 | 0.120 | -0.002 | 0.393 | 0.002 | 0.554 | 0.001 | 0.823 | 0.059 | 0.142 | 0.004 | 0.148 | 0.001 |
| Logical memory I total | 0.325 | 0.384 | 0.825 | 0.001 | 0.594 | 0.001 | 0.455 | 0.001 | 0.325 | 0.001 | 0.660 | 0.001 | 0.067 | -0.001 | 0.867 | 0.014 | 0.353 | -0.001 | 0.970 | 0.001 |
| Logical memory II total | 0.330 | 0.400 | 0.634 | 0.001 | 0.709 | 0.001 | 0.422 | 0.001 | 0.414 | 0.001 | 0.938 | 0.001 | 0.201 | -0.001 | 0.579 | 0.050 | 0.394 | -0.001 | 0.696 | 0.001 |
| Logical memory II recognition total | 0.708 | -0.381 | 0.582 | -0.001 | 0.305 | 0.001 | 0.637 | -0.001 | 0.707 | 0.001 | 0.066 | 0.003 | 0.444 | -0.001 | 0.549 | 0.133 | 0.588 | 0.001 | 0.202 | 0.001 |
| Trail Making Test A | 0.634 | 8.844 | 0.568 | 0.013 | 0.736 | -0.005 | 0.673 | 0.011 | 0.728 | 0.006 | 0.851 | -0.005 | 0.653 | 0.014 | 0.598 | 2.143 | 0.536 | -0.024 | 0.629 | -0.005 |
| Trail Making Test B | 0.599 | -3.189 | 0.986 | 0.001 | 0.961 | 0.001 | 0.520 | -0.006 | 0.587 | -0.003 | 0.578 | -0.005 | 0.147 | 0.015 | 0.458 | -0.986 | 0.592 | 0.007 | 0.225 | -0.004 |
| Digit coding score | 0.400 | -0.133 | 0.652 | 0.001 | 0.506 | 0.001 | 0.528 | 0.001 | 0.491 | 0.001 | 0.757 | 0.001 | 0.442 | 0.001 | 0.929 | 0.003 | 0.401 | 0.001 | 0.613 | 0.001 |
| Vocabulary | 0.070 | -0.483 | 0.017 | -0.001 | 0.084 | 0.001 | 0.025 | -0.001 | 0.062 | 0.001 | 0.309 | 0.001 | 0.183 | -0.001 | 0.344 | 0.056 | 0.067 | 0.001 | 0.895 | 0.001 |
| Word reading | 0.068 | -0.212 | 0.056 | -0.216 | 0.124 | 0.165 | 0.058 | -0.218 | 0.064 | -0.215 | 0.709 | 0.092 | 0.884 | 0.001 | 0.070 | 0.137 | 0.074 | 0.208 | 0.572 | 0.075 |
| EC: Effective connectivity, M: Male, F: Female, WA: White American, AA: African American, SBP: Systolic blood pressure, DBP: Diastolic blood pressure, WHM: White matter hyperintensities. | | | | | | | | | | | | | | | | | | | | |

**Table S2** Linear relationships between demographic, cardiometabolic, and cognitive measures and graph metrics derived from FC for Stroop task fMRI.

|  | Degree | | Clustering coefficient | | Modularity | | Transitivity | | Global efficiency | | Assortativity in-out | | Small- worldness | | Strength | | Characteristic path length | | Flow coefficient | |
| --- | --- | --- | --- | --- | --- | --- | --- | --- | --- | --- | --- | --- | --- | --- | --- | --- | --- | --- | --- | --- |
|  | p | β | p | β | p | β | p | β | p | β | p | β | p | β | p | β | p | β | p | β |
| Age at MRI | 0.081 | 0.424 | 0.061 | 0.001 | 0.674 | 0.001 | 0.214 | 0.001 | 0.055 | 0.001 | 0.604 | -0.001 | 0.364 | 0.002 | 0.730 | 0.008 | 0.337 | -0.001 | 0.135 | -0.001 |
| Gender  (M=0, F=1) | 0.093 | 4.086 | 0.284 | 0.007 | 0.062 | -0.014 | 0.053 | 0.010 | 0.272 | 0.006 | 0.203 | -0.022 | 0.487 | 0.013 | 0.936 | 0.019 | 0.076 | -0.023 | 0.047 | -0.014 |
| Race  (WA=0, AA=1) | 0.935 | 0.238 | 0.839 | 0.002 | 0.214 | 0.012 | 0.933 | -0.001 | 0.737 | 0.002 | 0.759 | 0.006 | 0.542 | 0.013 | 0.456 | 0.219 | 0.707 | 0.006 | 0.998 | 0.001 |
| Education | 0.415 | -10.16 | 0.901 | 0.004 | 0.404 | 0.035 | 0.820 | -0.006 | 0.598 | -0.017 | 0.125 | 0.126 | 0.728 | 0.060 | 0.119 | 1.976 | 0.353 | 0.062 | 0.607 | 0.019 |
| Smoking (non-smoker = 0, smoker = 1) | 0.437 | 2.623 | 0.615 | -0.005 | 0.539 | 0.007 | 0.931 | 0.001 | 0.294 | 0.009 | 0.540 | 0.015 | 0.680 | 0.021 | 0.529 | 0.234 | 0.426 | -0.014 | 0.725 | 0.004 |
| Alcohol  (non-drinker = 0, drinker = 1) | 0.263 | -6.987 | 0.626 | -0.009 | 0.690 | 0.009 | 0.403 | -0.011 | 0.446 | -0.012 | 0.646 | 0.020 | 0.975 | 0.003 | 0.645 | -0.316 | 0.312 | 0.033 | 0.363 | 0.019 |
| SBP | 0.494 | -0.054 | 0.481 | 0.001 | 0.704 | 0.001 | 0.576 | 0.001 | 0.554 | 0.001 | 0.768 | 0.001 | 0.578 | 0.001 | 0.702 | -0.003 | 0.650 | 0.001 | 0.404 | 0.001 |
| DBP | 0.976 | 0.004 | 0.629 | 0.001 | 0.862 | 0.001 | 0.970 | 0.001 | 0.878 | 0.001 | 0.917 | 0.001 | 0.665 | 0.001 | 0.347 | -0.012 | 0.751 | 0.001 | 0.769 | 0.001 |
| APOE-ε4 | 0.942 | 0.206 | 0.282 | -0.008 | 0.411 | 0.007 | 0.131 | -0.009 | 0.767 | 0.002 | 0.778 | 0.006 | 0.026 | -0.046 | 0.032 | 0.600 | 0.701 | 0.006 | 0.129 | 0.013 |
| BMI | 0.865 | 0.033 | 0.453 | 0.001 | 0.814 | 0.001 | 0.714 | 0.001 | 0.805 | 0.001 | 0.802 | 0.001 | 0.597 | -0.001 | 0.474 | 0.014 | 0.773 | 0.001 | 0.658 | 0.001 |
| Hemoglobin A1c | 0.458 | 0.787 | 0.864 | 0.001 | 0.486 | -0.002 | 0.773 | 0.001 | 0.424 | 0.002 | 0.544 | -0.004 | 0.641 | 0.003 | 0.029 | 0.223 | 0.390 | -0.005 | 0.595 | -0.002 |
| Fasting glucose | 0.894 | 0.007 | 0.657 | 0.001 | 0.808 | 0.001 | 0.583 | 0.001 | 0.631 | 0.001 | 0.653 | 0.001 | 0.933 | 0.001 | 0.573 | 0.003 | 0.745 | 0.001 | 0.685 | 0.001 |
| HOMA-IR | 0.920 | -0.032 | 0.520 | -0.001 | 0.592 | -0.001 | 0.760 | 0.001 | 0.680 | 0.001 | 0.931 | 0.001 | 0.949 | 0.001 | 0.635 | 0.016 | 0.789 | 0.001 | 0.991 | 0.001 |
| Fasting insulin | 0.792 | 0.050 | 0.153 | -0.001 | 0.306 | 0.001 | 0.284 | 0.001 | 0.459 | 0.001 | 0.634 | -0.001 | 0.153 | -0.002 | 0.268 | 0.023 | 0.865 | 0.001 | 0.177 | 0.001 |
| Gray matter volume | 0.720 | 27.34 | 0.802 | -0.055 | 0.999 | 0.001 | 0.982 | 0.004 | 0.867 | 0.032 | 0.058 | 1.052 | 0.258 | -0.685 | 0.167 | -10.83 | 0.663 | 0.179 | 0.634 | 0.114 |
| White matter volume | 0.053 | 163.5 | 0.495 | 0.154 | 0.235 | -0.310 | 0.128 | 0.248 | 0.101 | 0.316 | 0.006 | -1.537 | 0.498 | 0.421 | 0.303 | -8.294 | 0.060 | -1.059 | 0.055 | -0.465 |
| WMH volume | 0.094 | 4140 | 0.187 | -9.541 | 0.001 | -26.23 | 0.411 | 4.328 | 0.802 | 1.561 | 0.865 | -3.110 | 0.239 | -24.25 | 0.422 | -215.8 | 0.017 | -31.65 | 0.365 | -7.136 |
| Z-standardized mean score for all cognitive measures | 0.876 | -0.048 | 0.420 | -0.001 | 0.305 | 0.001 | 0.487 | 0.001 | 0.732 | 0.001 | 0.078 | 0.004 | 0.109 | -0.004 | 0.172 | -0.042 | 0.562 | 0.001 | 0.172 | 0.001 |
| Digit span forwards | 0.801 | -0.127 | 0.691 | -0.001 | 0.989 | 0.001 | 0.792 | 0.001 | 0.600 | -0.001 | 0.102 | 0.006 | 0.577 | -0.002 | 0.906 | 0.006 | 0.857 | 0.001 | 0.833 | 0.001 |
| Digit span backwards | 0.987 | 0.009 | 0.160 | -0.002 | 0.229 | 0.002 | 0.259 | -0.001 | 0.452 | 0.001 | 0.051 | 0.008 | 0.041 | -0.009 | 0.583 | -0.032 | 0.701 | 0.001 | 0.043 | 0.003 |
| Logical memory I total | 0.743 | -0.061 | 0.956 | 0.001 | 0.384 | 0.001 | 0.975 | 0.001 | 0.916 | 0.001 | 0.321 | 0.001 | 0.597 | -0.001 | 0.158 | -0.026 | 0.662 | 0.001 | 0.678 | 0.001 |
| Logical memory II total | 0.784 | 0.054 | 0.319 | -0.001 | 0.864 | 0.001 | 0.370 | 0.001 | 0.688 | 0.001 | 0.851 | 0.001 | 0.052 | -0.003 | 0.052 | -0.038 | 0.846 | 0.001 | 0.348 | 0.001 |
| Logical memory II recognition total | 0.927 | -0.045 | 0.611 | -0.001 | 0.927 | 0.001 | 0.718 | 0.001 | 0.955 | 0.001 | 0.063 | 0.006 | 0.191 | -0.005 | 0.183 | -0.064 | 0.514 | 0.002 | 0.376 | 0.001 |
| Trail Making Test A | 0.302 | 9.131 | 0.651 | -0.011 | 0.056 | -0.062 | 0.499 | 0.012 | 0.934 | 0.002 | 0.500 | -0.042 | 0.776 | -0.019 | 0.423 | 0.719 | 0.101 | -0.075 | 0.317 | -0.026 |
| Trail Making Test B | 0.455 | 2.163 | 0.182 | 0.010 | 0.825 | -0.002 | 0.184 | 0.008 | 0.500 | 0.005 | 0.486 | -0.014 | 0.146 | 0.031 | 0.945 | -0.020 | 0.513 | -0.010 | 0.150 | -0.012 |
| Digit coding score | 0.304 | 0.078 | 0.695 | 0.001 | 0.275 | 0.001 | 0.632 | 0.001 | 0.097 | 0.001 | 0.618 | 0.001 | 0.890 | 0.001 | 0.384 | -0.007 | 0.680 | 0.001 | 0.998 | 0.001 |
| Vocabulary | 0.786 | -0.035 | 0.734 | 0.001 | 0.682 | 0.001 | 0.381 | 0.001 | 0.937 | 0.001 | 0.483 | -0.001 | 0.621 | 0.001 | 0.147 | -0.018 | 0.735 | 0.001 | 0.476 | 0.001 |
| Word reading | 0.669 | -0.064 | 0.504 | 0.001 | 0.623 | 0.001 | 0.306 | 0.001 | 0.789 | 0.001 | 0.793 | 0.001 | 0.534 | -0.001 | 0.412 | -0.012 | 0.631 | 0.001 | 0.376 | 0.001 |
| FC: Functional connectivity, M: Male, F: Female, WA: White American, AA: African American, SBP: Systolic blood pressure, DBP: Diastolic blood pressure, WHM: White matter hyperintensities. | | | | | | | | | | | | | | | | | | | | |

**Table S3** Linear relationships between demographic, cardiometabolic, and cognitive measures and graph metrics derived from EC for resting-state fMRI.

|  | Degree | | Clustering coefficient | | Modularity | | Transitivity | | Global efficiency | | Assortativity in-out | | Small- worldness | | Strength | | Characteristic path length | | Flow coefficient | |
| --- | --- | --- | --- | --- | --- | --- | --- | --- | --- | --- | --- | --- | --- | --- | --- | --- | --- | --- | --- | --- |
|  | p | β | p | β | p | β | p | β | p | β | p | β | p | β | p | β | p | β | p | β |
| Age at MRI | 0.012 | -2.556 | 0.016 | -0.002 | 0.038 | 0.001 | 0.013 | -0.002 | 0.011 | -0.001 | 0.924 | 0.001 | 0.279 | 0.001 | 0.679 | 0.060 | 0.010 | 0.002 | 0.171 | 0.001 |
| Gender  (M=0, F=1) | 0.921 | -0.910 | 0.976 | 0.001 | 0.751 | 0.001 | 0.912 | -0.001 | 0.940 | 0.001 | 0.017 | -0.017 | 0.817 | 0.001 | 0.642 | 0.621 | 0.924 | 0.001 | 0.728 | -0.001 |
| Race  (WA=0, AA=1) | 0.266 | -12.55 | 0.418 | -0.008 | 0.242 | 0.004 | 0.350 | -0.009 | 0.259 | -0.006 | 0.657 | -0.004 | 0.680 | 0.003 | 0.068 | 3.025 | 0.263 | 0.012 | 0.912 | 0.001 |
| Education | 0.632 | 22.61 | 0.561 | 0.023 | 0.662 | 0.006 | 0.567 | 0.023 | 0.630 | 0.011 | 0.656 | -0.014 | 0.894 | 0.004 | 0.766 | -1.875 | 0.632 | -0.021 | 0.841 | 0.002 |
| Smoking (non-smoker = 0, smoker = 1) | 0.578 | 7.419 | 0.880 | 0.002 | 0.413 | -0.003 | 0.823 | 0.003 | 0.586 | 0.003 | 0.406 | 0.007 | 0.019 | -0.019 | 0.392 | -1.456 | 0.611 | -0.006 | 0.611 | -0.002 |
| Alcohol  (non-drinker = 0, drinker = 1) | 0.995 | 0.170 | 0.771 | -0.006 | 0.975 | 0.001 | 0.770 | -0.006 | 0.993 | 0.001 | 0.999 | 0.001 | 0.015 | -0.036 | 0.860 | -0.560 | 0.966 | -0.001 | 0.695 | -0.002 |
| SBP | 0.240 | -13.25 | 0.373 | -0.009 | 0.220 | 0.004 | 0.315 | -0.010 | 0.235 | -0.006 | 0.702 | -0.003 | 0.736 | 0.002 | 0.073 | 2.991 | 0.240 | 0.013 | 0.865 | 0.001 |
| DBP | 0.239 | -13.40 | 0.359 | -0.009 | 0.206 | 0.004 | 0.306 | -0.010 | 0.236 | -0.006 | 0.716 | -0.003 | 0.802 | 0.002 | 0.080 | 2.922 | 0.240 | 0.013 | 0.874 | 0.001 |
| APOE-ε4 | 0.259 | -14.64 | 0.327 | -0.011 | 0.296 | 0.004 | 0.291 | -0.012 | 0.253 | -0.007 | 0.372 | -0.009 | 0.629 | 0.004 | 0.092 | 3.235 | 0.248 | 0.014 | 0.804 | -0.001 |
| BMI | 0.332 | -11.66 | 0.527 | -0.007 | 0.377 | 0.003 | 0.441 | -0.008 | 0.328 | -0.006 | 0.669 | -0.004 | 0.351 | 0.007 | 0.015 | 4.167 | 0.334 | 0.011 | 0.969 | 0.001 |
| Hemoglobin A1c | 0.507 | 2.622 | 0.434 | 0.003 | 0.385 | -0.001 | 0.416 | 0.003 | 0.524 | 0.001 | 0.449 | 0.002 | 0.180 | 0.003 | 0.500 | 0.388 | 0.535 | -0.002 | 0.899 | 0.001 |
| Fasting glucose | 0.263 | -12.85 | 0.422 | -0.008 | 0.212 | 0.004 | 0.358 | -0.009 | 0.255 | -0.006 | 0.775 | -0.002 | 0.637 | 0.003 | 0.035 | 3.475 | 0.260 | 0.012 | 0.957 | 0.001 |
| HOMA-IR | 0.538 | -12.05 | 0.552 | -0.010 | 0.310 | 0.007 | 0.474 | -0.012 | 0.525 | -0.006 | 0.179 | -0.017 | 0.323 | -0.012 | 0.003 | 7.011 | 0.526 | 0.012 | 0.928 | 0.001 |
| Fasting insulin | 0.491 | -13.59 | 0.495 | -0.011 | 0.298 | 0.007 | 0.423 | -0.014 | 0.479 | -0.007 | 0.175 | -0.018 | 0.319 | -0.013 | 0.004 | 7.033 | 0.481 | 0.013 | 0.950 | 0.001 |
| Gray matter volume | 0.311 | -11.80 | 0.465 | -0.007 | 0.290 | 0.004 | 0.394 | -0.009 | 0.303 | -0.006 | 0.442 | -0.006 | 0.675 | 0.003 | 0.057 | 3.910 | 0.309 | 0.011 | 0.980 | 0.001 |
| White matter volume | 0.355 | -10.86 | 0.543 | -0.006 | 0.318 | 0.004 | 0.463 | -0.007 | 0.346 | -0.005 | 0.469 | -0.006 | 0.540 | 0.004 | 0.058 | 3.880 | 0.354 | 0.010 | 0.944 | 0.001 |
| WMH volume | 0.497 | -8.214 | 0.631 | -0.005 | 0.416 | 0.003 | 0.560 | -0.006 | 0.489 | -0.004 | 0.435 | -0.006 | 0.758 | 0.002 | 0.025 | 3.125 | 0.498 | 0.008 | 0.719 | -0.001 |
| Z-standardized mean score for all cognitive measures | 0.282 | -12.30 | 0.463 | -0.007 | 0.296 | 0.004 | 0.388 | -0.009 | 0.275 | -0.006 | 0.614 | -0.004 | 0.554 | 0.004 | 0.061 | 3.149 | 0.278 | 0.012 | 0.945 | 0.001 |
| Digit span forwards | 0.269 | -12.52 | 0.417 | -0.008 | 0.244 | 0.004 | 0.351 | -0.009 | 0.263 | -0.006 | 0.669 | -0.004 | 0.704 | 0.003 | 0.070 | 3.005 | 0.266 | 0.012 | 0.908 | 0.001 |
| Digit span backwards | 0.267 | -12.58 | 0.421 | -0.008 | 0.241 | 0.004 | 0.353 | -0.009 | 0.260 | -0.006 | 0.642 | -0.004 | 0.678 | 0.003 | 0.069 | 3.030 | 0.264 | 0.012 | 0.907 | 0.001 |
| Logical memory I total | 0.268 | -12.58 | 0.424 | -0.008 | 0.257 | 0.004 | 0.356 | -0.009 | 0.261 | -0.006 | 0.693 | -0.003 | 0.626 | 0.003 | 0.071 | 3.012 | 0.265 | 0.012 | 0.902 | 0.001 |
| Logical memory II total | 0.310 | -11.79 | 0.425 | -0.008 | 0.407 | 0.003 | 0.368 | -0.009 | 0.302 | -0.006 | 0.763 | -0.003 | 0.843 | 0.001 | 0.044 | 3.416 | 0.303 | 0.011 | 0.937 | 0.001 |
| Logical memory II recognition total | 0.255 | -13.01 | 0.399 | -0.008 | 0.247 | 0.004 | 0.332 | -0.010 | 0.249 | -0.006 | 0.643 | -0.004 | 0.732 | 0.002 | 0.055 | 3.202 | 0.250 | 0.012 | 0.934 | 0.001 |
| Trail Making Test A | 0.275 | -12.46 | 0.438 | -0.008 | 0.300 | 0.004 | 0.368 | -0.009 | 0.268 | -0.006 | 0.662 | -0.004 | 0.650 | 0.003 | 0.092 | 2.806 | 0.274 | 0.012 | 0.907 | 0.001 |
| Trail Making Test B | 0.319 | -11.30 | 0.508 | -0.006 | 0.283 | 0.004 | 0.425 | -0.008 | 0.311 | -0.006 | 0.611 | -0.004 | 0.616 | 0.003 | 0.065 | 3.095 | 0.316 | 0.011 | 0.946 | 0.001 |
| Digit coding score | 0.280 | -12.31 | 0.457 | -0.007 | 0.272 | 0.004 | 0.384 | -0.009 | 0.272 | -0.006 | 0.632 | -0.004 | 0.570 | 0.004 | 0.068 | 3.049 | 0.275 | 0.012 | 0.973 | 0.001 |
| Vocabulary | 0.452 | -8.616 | 0.650 | -0.004 | 0.478 | 0.002 | 0.557 | -0.006 | 0.443 | -0.004 | 0.472 | -0.006 | 0.422 | 0.006 | 0.055 | 3.278 | 0.447 | 0.008 | 0.974 | 0.001 |
| Word reading | 0.313 | -11.53 | 0.489 | -0.007 | 0.272 | 0.004 | 0.412 | -0.008 | 0.303 | -0.006 | 0.587 | -0.005 | 0.589 | 0.004 | 0.038 | 3.446 | 0.306 | 0.011 | 0.976 | 0.001 |
| EC: Effective connectivity, M: Male, F: Female, WA: White American, AA: African American, SBP: Systolic blood pressure, DBP: Diastolic blood pressure, WHM: White matter hyperintensities. | | | | | | | | | | | | | | | | | | | | |

**Table S4** Linear relationships between demographic, cardiometabolic, and cognitive measures and graph metrics derived from FC for resting-state fMRI.

|  | Degree | | Clustering coefficient | | Modularity | | Transitivity | | Global efficiency | | Assortativity in-out | | Small- worldness | | Strength | | Characteristic path length | | Flow coefficient | |
| --- | --- | --- | --- | --- | --- | --- | --- | --- | --- | --- | --- | --- | --- | --- | --- | --- | --- | --- | --- | --- |
|  | p | β | p | β | p | β | p | β | p | β | p | β | p | β | p | β | p | β | p | β |
| Age at MRI | 0.097 | 0.774 | 0.474 | -0.001 | 0.404 | -0.001 | 0.945 | 0.001 | 0.142 | 0.001 | 0.503 | 0.001 | 0.195 | -0.004 | 0.425 | 0.034 | 0.209 | -0.003 | 0.889 | 0.001 |
| Gender  (M=0, F=1) | 0.294 | -4.482 | 0.552 | 0.005 | 0.693 | 0.004 | 0.994 | 0.001 | 0.305 | -0.007 | 0.676 | -0.007 | 0.857 | -0.005 | 0.004 | -1.145 | 0.325 | 0.023 | 0.791 | 0.002 |
| Race  (WA=0, AA=1) | 0.416 | 4.260 | 0.697 | -0.004 | 0.775 | -0.004 | 0.738 | -0.002 | 0.422 | 0.006 | 0.298 | 0.023 | 0.321 | 0.031 | 0.567 | 0.275 | 0.417 | -0.023 | 0.639 | -0.003 |
| Education | 0.958 | 1.135 | 0.808 | 0.009 | 0.666 | 0.025 | 0.349 | 0.017 | 0.895 | 0.004 | 0.609 | -0.040 | 0.703 | 0.040 | 0.751 | 0.583 | 0.861 | -0.020 | 0.610 | -0.014 |
| Smoking (non-smoker = 0, smoker = 1) | 0.577 | -3.331 | 0.470 | -0.009 | 0.729 | 0.006 | 0.077 | -0.010 | 0.773 | -0.003 | 0.580 | -0.014 | 0.769 | -0.010 | 0.926 | -0.050 | 0.917 | 0.004 | 0.250 | 0.009 |
| Alcohol  (non-drinker = 0, drinker = 1) | 0.885 | -1.605 | 0.906 | -0.003 | 0.760 | -0.010 | 0.117 | -0.016 | 0.992 | 0.001 | 0.497 | -0.031 | 0.689 | 0.024 | 0.009 | 2.522 | 0.920 | -0.007 | 0.843 | 0.003 |
| SBP | 0.439 | 4.066 | 0.663 | -0.004 | 0.762 | -0.004 | 0.633 | -0.002 | 0.442 | 0.006 | 0.312 | 0.022 | 0.373 | 0.028 | 0.570 | 0.276 | 0.435 | -0.023 | 0.715 | -0.003 |
| DBP | 0.446 | 4.036 | 0.638 | -0.005 | 0.753 | -0.004 | 0.558 | -0.003 | 0.442 | 0.006 | 0.321 | 0.022 | 0.443 | 0.024 | 0.579 | 0.270 | 0.433 | -0.023 | 0.775 | -0.002 |
| APOE-ε4 | 0.155 | 8.468 | 0.156 | -0.016 | 0.422 | -0.012 | 0.297 | -0.006 | 0.142 | 0.013 | 0.666 | 0.011 | 0.808 | 0.009 | 0.669 | 0.230 | 0.137 | -0.048 | 0.860 | 0.001 |
| BMI | 0.355 | 5.223 | 0.931 | 0.001 | 0.604 | -0.007 | 0.921 | 0.001 | 0.360 | 0.008 | 0.522 | 0.015 | 0.126 | 0.051 | 0.963 | -0.024 | 0.358 | -0.028 | 0.257 | -0.008 |
| Hemoglobin A1c | 0.452 | 1.386 | 0.610 | 0.002 | 0.134 | -0.005 | 0.066 | 0.003 | 0.504 | 0.002 | 0.318 | 0.008 | 0.243 | 0.012 | 0.453 | -0.128 | 0.537 | -0.006 | 0.128 | -0.004 |
| Fasting glucose | 0.500 | 3.617 | 0.772 | -0.003 | 0.860 | -0.002 | 0.708 | -0.002 | 0.510 | 0.005 | 0.355 | 0.020 | 0.332 | 0.031 | 0.656 | 0.221 | 0.506 | -0.020 | 0.666 | -0.003 |
| HOMA-IR | 0.327 | 9.762 | 0.680 | -0.008 | 0.602 | -0.015 | 0.352 | -0.008 | 0.379 | 0.014 | 0.416 | 0.027 | 0.879 | -0.008 | 0.649 | 0.403 | 0.423 | -0.045 | 0.899 | -0.002 |
| Fasting insulin | 0.325 | 9.916 | 0.704 | -0.007 | 0.588 | -0.016 | 0.372 | -0.008 | 0.383 | 0.014 | 0.418 | 0.028 | 0.881 | -0.008 | 0.720 | 0.317 | 0.433 | -0.045 | 0.877 | -0.002 |
| Gray matter volume | 0.145 | 6.729 | 0.566 | -0.006 | 0.633 | -0.006 | 0.745 | -0.002 | 0.176 | 0.010 | 0.533 | 0.014 | 0.325 | 0.032 | 0.383 | 0.420 | 0.197 | -0.036 | 0.512 | -0.004 |
| White matter volume | 0.140 | 6.799 | 0.521 | -0.006 | 0.638 | -0.006 | 0.765 | -0.001 | 0.168 | 0.010 | 0.522 | 0.014 | 0.346 | 0.031 | 0.380 | 0.422 | 0.186 | -0.037 | 0.541 | -0.004 |
| WMH volume | 0.224 | 5.675 | 0.382 | -0.009 | 0.770 | -0.004 | 0.502 | -0.003 | 0.267 | 0.008 | 0.356 | 0.021 | 0.627 | 0.016 | 0.373 | 0.444 | 0.294 | -0.029 | 0.960 | 0.001 |
| Z-standardized mean score for all cognitive measures | 0.369 | 4.762 | 0.769 | -0.003 | 0.830 | -0.003 | 0.842 | -0.001 | 0.368 | 0.007 | 0.403 | 0.018 | 0.290 | 0.034 | 0.582 | 0.269 | 0.359 | -0.027 | 0.519 | -0.005 |
| Digit span forwards | 0.417 | 4.270 | 0.691 | -0.004 | 0.783 | -0.004 | 0.742 | -0.002 | 0.424 | 0.006 | 0.291 | 0.023 | 0.326 | 0.031 | 0.564 | 0.279 | 0.420 | -0.023 | 0.646 | -0.003 |
| Digit span backwards | 0.419 | 4.258 | 0.703 | -0.004 | 0.781 | -0.004 | 0.742 | -0.002 | 0.425 | 0.006 | 0.300 | 0.023 | 0.321 | 0.032 | 0.561 | 0.279 | 0.421 | -0.023 | 0.635 | -0.003 |
| Logical memory I total | 0.412 | 4.321 | 0.724 | -0.003 | 0.790 | -0.004 | 0.769 | -0.001 | 0.417 | 0.007 | 0.326 | 0.021 | 0.316 | 0.032 | 0.595 | 0.257 | 0.412 | -0.024 | 0.606 | -0.004 |
| Logical memory II total | 0.315 | 5.397 | 0.715 | -0.004 | 0.769 | -0.004 | 0.974 | 0.001 | 0.322 | 0.008 | 0.446 | 0.017 | 0.288 | 0.035 | 0.607 | 0.255 | 0.323 | -0.029 | 0.509 | -0.005 |
| Logical memory II recognition total | 0.426 | 4.220 | 0.845 | -0.002 | 0.825 | -0.003 | 0.876 | -0.001 | 0.436 | 0.006 | 0.377 | 0.019 | 0.237 | 0.037 | 0.669 | 0.207 | 0.432 | -0.023 | 0.494 | -0.005 |
| Trail Making Test A | 0.511 | 3.455 | 0.722 | -0.004 | 0.966 | -0.001 | 0.704 | -0.002 | 0.503 | 0.005 | 0.348 | 0.021 | 0.330 | 0.031 | 0.647 | 0.222 | 0.486 | -0.020 | 0.692 | -0.003 |
| Trail Making Test B | 0.251 | 5.834 | 0.601 | -0.005 | 0.655 | -0.006 | 0.783 | -0.001 | 0.249 | 0.009 | 0.315 | 0.022 | 0.349 | 0.030 | 0.508 | 0.321 | 0.247 | -0.032 | 0.571 | -0.004 |
| Digit coding score | 0.394 | 4.507 | 0.720 | -0.004 | 0.837 | -0.003 | 0.779 | -0.001 | 0.387 | 0.007 | 0.360 | 0.020 | 0.320 | 0.032 | 0.520 | 0.312 | 0.373 | -0.026 | 0.594 | -0.004 |
| Vocabulary | 0.327 | 5.279 | 0.740 | -0.003 | 0.847 | -0.003 | 0.858 | -0.001 | 0.319 | 0.008 | 0.492 | 0.015 | 0.228 | 0.039 | 0.599 | 0.261 | 0.308 | -0.030 | 0.478 | -0.005 |
| Word reading | 0.329 | 5.161 | 0.660 | -0.004 | 0.749 | -0.004 | 0.760 | -0.001 | 0.327 | 0.008 | 0.390 | 0.019 | 0.320 | 0.032 | 0.463 | 0.357 | 0.320 | -0.029 | 0.583 | -0.004 |
| FC: Functional connectivity, M: Male, F: Female, WA: White American, AA: African American, SBP: Systolic blood pressure, DBP: Diastolic blood pressure, WHM: White matter hyperintensities. | | | | | | | | | | | | | | | | | | | | |

**Table S5** Linear relationships between demographic, cardiometabolic, and cognitive measures and graph metrics derived from EC among 24 task-related ROIs for Stroop task fMRI and eight core DMN ROIs for Stroop task fMRI.

|  | Degree | | Clustering coefficient | | Modularity | | Transitivity | | Global efficiency | | Assortativity in-out | | Small- worldness | | Strength | | Characteristic path length | | Flow coefficient | |
| --- | --- | --- | --- | --- | --- | --- | --- | --- | --- | --- | --- | --- | --- | --- | --- | --- | --- | --- | --- | --- |
|  | p | β | p | β | p | β | p | β | p | β | p | β | p | β | p | β | p | β | p | β |
| Age at MRI | 0.651 | 0.468 | 0.516 | 0.001 | 0.441 | 0.001 | 0.591 | 0.001 | 0.644 | 0.001 | 0.151 | -0.001 | 0.367 | 0.001 | 0.841 | -0.036 | 0.631 | 0.001 | 0.926 | 0.001 |
| Gender  (M=0, F=1) | 0.615 | 4.790 | 0.706 | 0.003 | 0.452 | 0.002 | 0.586 | 0.005 | 0.642 | 0.002 | 0.043 | 0.002 | 0.653 | -0.002 | 0.466 | 1.192 | 0.636 | -0.005 | 0.718 | 0.001 |
| Race  (WA=0, AA=1) | 0.462 | -8.600 | 0.613 | -0.005 | 0.601 | 0.002 | 0.594 | -0.005 | 0.462 | -0.004 | 0.023 | 0.008 | 0.812 | -0.002 | 0.049 | 3.947 | 0.475 | 0.008 | 0.663 | -0.001 |
| Education | 0.769 | -14.17 | 0.865 | -0.007 | 0.274 | -0.015 | 0.844 | -0.009 | 0.779 | -0.007 | 0.521 | -0.019 | 0.908 | 0.003 | 0.493 | -4.776 | 0.776 | 0.014 | 0.931 | 0.001 |
| Smoking (non-smoker = 0, smoker = 1) | 0.992 | -0.146 | 0.896 | -0.002 | 0.766 | 0.001 | 0.875 | -0.002 | 0.960 | 0.001 | 0.257 | 0.011 | 0.169 | -0.009 | 0.671 | -0.756 | 0.946 | -0.001 | 0.817 | 0.001 |
| Alcohol  (non-drinker = 0, drinker = 1) | 0.609 | -13.24 | 0.539 | -0.014 | 0.982 | 0.001 | 0.558 | -0.013 | 0.596 | -0.007 | 0.955 | 0.001 | 0.100 | -0.020 | 0.262 | -3.685 | 0.589 | 0.014 | 0.801 | -0.002 |
| SBP | 0.059 | -0.309 | 0.255 | 0.001 | 0.015 | 0.001 | 0.199 | 0.001 | 0.060 | 0.001 | 0.931 | 0.001 | 0.020 | 0.001 | 0.392 | 0.031 | 0.039 | 0.001 | 0.364 | 0.001 |
| DBP | 0.001 | -0.845 | 0.006 | -0.001 | 0.008 | 0.001 | 0.003 | -0.001 | 0.002 | -0.001 | 0.516 | 0.001 | 0.120 | 0.001 | 0.273 | 0.066 | 0.001 | 0.001 | 0.440 | 0.001 |
| APOE-ε4 | 0.878 | -1.743 | 0.875 | -0.002 | 0.986 | 0.001 | 0.920 | -0.001 | 0.875 | -0.001 | 1.000 | 0.001 | 0.655 | -0.003 | 0.125 | -2.841 | 0.865 | 0.002 | 0.449 | 0.002 |
| BMI | 0.605 | 0.397 | 0.767 | 0.001 | 0.124 | 0.001 | 0.709 | 0.001 | 0.598 | 0.001 | 0.352 | 0.001 | 0.997 | 0.001 | 0.087 | -0.215 | 0.590 | 0.001 | 0.031 | 0.001 |
| Hemoglobin A1c | 0.832 | -0.873 | 0.752 | -0.001 | 0.650 | 0.001 | 0.755 | -0.001 | 0.803 | -0.001 | 0.169 | -0.004 | 0.725 | -0.001 | 0.447 | 0.536 | 0.765 | 0.001 | 0.127 | -0.001 |
| Fasting glucose | 0.071 | -0.380 | 0.042 | 0.001 | 0.081 | 0.001 | 0.046 | 0.001 | 0.064 | 0.001 | 0.041 | 0.001 | 0.584 | 0.001 | 0.957 | -0.002 | 0.055 | 0.001 | 0.737 | 0.001 |
| HOMA-IR | 0.042 | -4.976 | 0.043 | -0.004 | 0.061 | 0.001 | 0.051 | -0.004 | 0.039 | -0.003 | 0.715 | -0.001 | 0.124 | 0.002 | 0.673 | -0.119 | 0.037 | 0.005 | 0.367 | 0.001 |
| Fasting insulin | 0.188 | -0.979 | 0.242 | -0.001 | 0.180 | 0.001 | 0.282 | -0.001 | 0.186 | -0.001 | 0.192 | 0.001 | 0.019 | 0.001 | 0.937 | -0.007 | 0.192 | 0.001 | 0.459 | 0.001 |
| Gray matter volume | 0.991 | -0.803 | 0.884 | -0.010 | 0.718 | -0.008 | 0.876 | -0.010 | 0.995 | 0.001 | 0.600 | -0.030 | 0.225 | 0.049 | 0.086 | 19.442 | 0.992 | 0.001 | 0.508 | 0.011 |
| White matter volume | 0.951 | -2.718 | 0.860 | -0.007 | 0.176 | -0.018 | 0.824 | -0.009 | 0.963 | -0.001 | 0.811 | -0.008 | 0.903 | -0.003 | 0.311 | 7.388 | 0.951 | 0.003 | 0.076 | -0.017 |
| WMH volume | 0.536 | 177.8 | 0.503 | 0.168 | 0.445 | -0.068 | 0.491 | 0.175 | 0.528 | 0.092 | 0.330 | 0.191 | 0.416 | -0.138 | 0.688 | -17.64 | 0.519 | -0.186 | 0.207 | 0.086 |
| Z-standardized mean score for all cognitive measures | 0.724 | 111.4 | 0.709 | 0.102 | 0.762 | 0.030 | 0.656 | 0.124 | 0.718 | 0.058 | 0.168 | 0.295 | 0.265 | 0.208 | 0.681 | -19.84 | 0.698 | -0.122 | 0.994 | -0.001 |
| Digit span forwards | 0.778 | 2765 | 0.671 | 3.635 | 0.945 | -0.205 | 0.731 | 2.978 | 0.761 | 1.513 | 0.546 | -4.095 | 0.595 | 3.129 | 0.045 | 2782 | 0.768 | -2.898 | 0.390 | -1.962 |
| Digit span backwards | 0.931 | -0.106 | 0.935 | 0.001 | 0.613 | 0.001 | 0.938 | 0.001 | 0.973 | 0.001 | 0.621 | 0.001 | 0.252 | 0.001 | 0.612 | -0.108 | 0.997 | 0.001 | 0.403 | 0.001 |
| Logical memory I total | 0.523 | 1.262 | 0.389 | 0.001 | 0.865 | 0.001 | 0.362 | 0.002 | 0.488 | 0.001 | 0.013 | 0.003 | 0.558 | 0.001 | 0.691 | -0.135 | 0.464 | -0.001 | 0.433 | 0.001 |
| Logical memory II total | 0.159 | -3.142 | 0.216 | -0.002 | 0.370 | 0.001 | 0.226 | -0.002 | 0.175 | -0.002 | 0.790 | 0.001 | 0.638 | 0.001 | 0.664 | -0.167 | 0.183 | 0.003 | 0.029 | -0.001 |
| Logical memory II recognition total | 0.632 | 0.358 | 0.640 | 0.001 | 0.613 | 0.001 | 0.669 | 0.001 | 0.605 | 0.001 | 0.486 | 0.001 | 0.919 | 0.001 | 0.499 | -0.087 | 0.596 | 0.001 | 0.971 | 0.001 |
| Trail Making Test A | 0.354 | 0.735 | 0.415 | 0.001 | 0.794 | 0.001 | 0.429 | 0.001 | 0.345 | 0.001 | 0.557 | 0.001 | 0.869 | 0.001 | 0.951 | 0.008 | 0.342 | -0.001 | 0.484 | 0.001 |
| Trail Making Test B | 0.801 | -0.485 | 0.802 | 0.001 | 0.361 | 0.001 | 0.766 | -0.001 | 0.839 | 0.001 | 0.724 | 0.001 | 0.507 | 0.001 | 0.545 | -0.200 | 0.843 | 0.001 | 0.708 | 0.001 |
| Digit coding score | 0.528 | 22.68 | 0.614 | 0.016 | 0.798 | -0.003 | 0.629 | 0.015 | 0.541 | 0.011 | 0.761 | 0.007 | 0.319 | -0.020 | 0.539 | 3.786 | 0.552 | -0.021 | 0.096 | -0.014 |
| Vocabulary | 0.982 | 0.256 | 0.961 | 0.001 | 0.715 | -0.001 | 0.940 | 0.001 | 0.994 | 0.001 | 0.583 | -0.004 | 0.968 | 0.001 | 0.994 | 0.014 | 0.989 | 0.001 | 0.851 | 0.001 |
| Word reading | 0.701 | -0.116 | 0.993 | 0.001 | 0.293 | 0.001 | 0.983 | 0.001 | 0.706 | 0.001 | 0.526 | 0.001 | 0.010 | 0.001 | 0.910 | 0.006 | 0.713 | 0.001 | 0.805 | 0.001 |
| EC: Effective connectivity, M: Male, F: Female, WA: White American, AA: African American, SBP: Systolic blood pressure, DBP: Diastolic blood pressure, WHM: White matter hyperintensities. | | | | | | | | | | | | | | | | | | | | |

**Table S6** Linear relationships between demographic, cardiometabolic, and cognitive measures and graph metrics derived from FC among 24 task-related ROIs for Stroop task fMRI and eight core DMN ROIs for Stroop task fMRI.

|  | Degree | | Clustering coefficient | | Modularity | | Transitivity | | Global efficiency | | Assortativity in-out | | Small- worldness | | Strength | | Characteristic path length | | Flow coefficient | |
| --- | --- | --- | --- | --- | --- | --- | --- | --- | --- | --- | --- | --- | --- | --- | --- | --- | --- | --- | --- | --- |
|  | p | β | p | β | p | β | p | β | p | β | p | β | p | β | p | β | p | β | p | β |
| Age at MRI | 0.465 | 0.317 | 0.933 | 0.001 | 0.724 | 0.001 | 0.392 | 0.001 | 0.553 | 0.001 | 0.148 | 0.002 | 0.543 | -0.009 | 0.075 | 0.061 | 0.561 | -0.001 | 0.624 | 0.001 |
| Gender  (M=0, F=1) | 0.587 | 2.166 | 0.211 | 0.007 | 0.306 | -0.008 | 0.116 | 0.006 | 0.845 | 0.001 | 0.810 | 0.003 | 0.525 | 0.082 | 0.448 | -0.237 | 0.636 | -0.006 | 0.141 | -0.008 |
| Race  (WA=0, AA=1) | 0.695 | -1.919 | 0.358 | -0.006 | 0.279 | 0.011 | 0.547 | -0.003 | 0.990 | 0.001 | 0.662 | -0.007 | 0.292 | -0.167 | 0.580 | 0.211 | 1.000 | 0.001 | 0.394 | 0.006 |
| Education | 0.436 | -16.10 | 0.327 | 0.025 | 0.416 | 0.037 | 0.965 | -0.001 | 0.508 | -0.018 | 0.456 | -0.048 | 0.789 | 0.164 | 0.658 | 0.681 | 0.362 | 0.059 | 0.775 | -0.008 |
| Smoking (non-smoker = 0, smoker = 1) | 0.276 | 5.731 | 0.764 | -0.002 | 0.856 | -0.002 | 0.883 | -0.001 | 0.186 | 0.009 | 0.098 | -0.028 | 0.840 | -0.037 | 0.386 | -0.384 | 0.186 | -0.022 | 0.945 | -0.001 |
| Alcohol  (non-drinker = 0, drinker = 1) | 0.784 | 2.697 | 0.634 | -0.007 | 0.688 | -0.009 | 0.836 | 0.002 | 0.812 | 0.003 | 0.168 | 0.043 | 0.265 | 0.376 | 0.990 | 0.010 | 0.929 | -0.003 | 0.794 | 0.004 |
| SBP | 0.885 | -0.019 | 0.513 | 0.001 | 0.177 | 0.001 | 0.238 | 0.001 | 0.845 | 0.001 | 0.053 | 0.001 | 0.248 | -0.005 | 0.619 | 0.005 | 0.838 | 0.001 | 0.738 | 0.001 |
| DBP | 0.757 | 0.068 | 0.786 | 0.001 | 0.512 | 0.001 | 0.409 | 0.001 | 0.580 | 0.001 | 0.286 | 0.001 | 0.502 | -0.005 | 0.886 | 0.002 | 0.761 | 0.001 | 0.719 | 0.001 |
| APOE-ε4 | 0.809 | 1.146 | 0.431 | -0.005 | 0.981 | 0.001 | 0.142 | -0.007 | 0.656 | 0.003 | 0.214 | -0.019 | 0.387 | 0.128 | 0.897 | 0.048 | 0.816 | -0.004 | 0.337 | 0.006 |
| BMI | 0.489 | 0.221 | 0.035 | -0.001 | 0.818 | 0.001 | 0.342 | 0.001 | 0.369 | 0.001 | 0.726 | 0.001 | 0.037 | 0.021 | 0.917 | 0.003 | 0.288 | -0.001 | 0.269 | 0.001 |
| Hemoglobin A1c | 0.221 | 2.095 | 0.885 | 0.001 | 0.822 | 0.001 | 0.354 | 0.002 | 0.187 | 0.003 | 0.413 | -0.005 | 0.453 | -0.040 | 0.536 | 0.083 | 0.183 | -0.007 | 0.354 | -0.002 |
| Fasting glucose | 0.664 | 0.038 | 0.862 | 0.001 | 0.497 | 0.001 | 0.602 | 0.001 | 0.510 | 0.001 | 0.600 | 0.001 | 0.496 | -0.002 | 0.604 | 0.004 | 0.579 | 0.001 | 0.691 | 0.001 |
| HOMA-IR | 0.266 | 1.076 | 0.704 | 0.001 | 0.639 | 0.001 | 0.295 | 0.001 | 0.167 | 0.002 | 0.951 | 0.001 | 0.225 | -0.041 | 0.132 | 0.122 | 0.243 | -0.004 | 0.486 | -0.001 |
| Fasting insulin | 0.423 | 0.233 | 0.770 | 0.001 | 0.684 | 0.001 | 0.386 | 0.001 | 0.255 | 0.001 | 0.837 | 0.001 | 0.346 | -0.009 | 0.225 | 0.030 | 0.379 | -0.001 | 0.665 | 0.001 |
| Gray matter volume | 0.656 | 50.55 | 0.847 | -0.032 | 0.377 | -0.227 | 0.447 | -0.096 | 0.759 | 0.046 | 0.155 | -0.561 | 0.942 | -0.295 | 0.101 | -15.77 | 0.678 | -0.155 | 0.910 | 0.019 |
| White matter volume | 0.665 | 53.87 | 0.078 | 0.313 | 0.660 | -0.124 | 0.030 | 0.296 | 0.905 | 0.020 | 0.299 | 0.450 | 0.779 | 1.238 | 0.489 | -7.367 | 0.806 | -0.100 | 0.036 | -0.387 |
| WMH volume | 0.353 | 3633 | 0.903 | 0.690 | 0.027 | -19.34 | 0.097 | 7.157 | 0.735 | 1.768 | 0.975 | 0.433 | 0.171 | -188.8 | 0.781 | 95.54 | 0.158 | -18.08 | 0.158 | -8.265 |
| Z-standardized mean score for all cognitive measures | 0.772 | 0.150 | 0.277 | 0.001 | 0.520 | 0.001 | 0.495 | 0.001 | 0.642 | 0.001 | 0.646 | -0.001 | 0.833 | -0.004 | 0.642 | 0.019 | 0.957 | 0.001 | 0.578 | 0.001 |
| Digit span forwards | 0.749 | 0.265 | 0.060 | 0.002 | 0.473 | -0.001 | 0.094 | 0.001 | 0.975 | 0.001 | 0.542 | -0.002 | 0.733 | 0.009 | 0.106 | 0.104 | 0.978 | 0.001 | 0.083 | -0.002 |
| Digit span backwards | 0.878 | -0.144 | 0.946 | 0.001 | 0.362 | 0.002 | 0.481 | -0.001 | 0.875 | 0.001 | 0.843 | 0.001 | 0.578 | 0.017 | 0.914 | 0.008 | 0.656 | 0.001 | 0.521 | 0.001 |
| Logical memory I total | 0.718 | -0.113 | 0.831 | 0.001 | 0.669 | 0.001 | 0.419 | 0.001 | 0.739 | 0.001 | 0.439 | -0.001 | 0.354 | 0.009 | 0.879 | 0.004 | 0.545 | 0.001 | 0.690 | 0.001 |
| Logical memory II total | 0.534 | 0.207 | 0.890 | 0.001 | 0.689 | 0.001 | 0.992 | 0.001 | 0.530 | 0.001 | 0.405 | -0.001 | 0.601 | -0.006 | 0.217 | -0.032 | 0.602 | -0.001 | 0.893 | 0.001 |
| Logical memory II recognition total | 0.638 | -0.378 | 0.340 | -0.001 | 0.388 | 0.001 | 0.506 | -0.001 | 0.851 | 0.001 | 0.951 | 0.001 | 0.938 | 0.002 | 0.707 | -0.024 | 0.781 | 0.001 | 0.247 | 0.001 |
| Trail Making Test A | 0.313 | 15.14 | 0.254 | -0.022 | 0.023 | -0.070 | 0.985 | 0.001 | 0.535 | 0.012 | 0.192 | -0.063 | 0.897 | 0.063 | 0.234 | -1.415 | 0.109 | -0.076 | 0.766 | -0.006 |
| Trail Making Test B | 0.764 | -1.422 | 0.379 | -0.005 | 0.837 | -0.002 | 0.225 | -0.006 | 0.792 | -0.002 | 0.604 | 0.008 | 0.466 | 0.111 | 0.662 | -0.161 | 0.860 | 0.003 | 0.356 | 0.006 |
| Digit coding score | 0.102 | 0.205 | 0.103 | 0.001 | 0.790 | 0.001 | 0.026 | 0.001 | 0.046 | 0.001 | 0.639 | 0.001 | 0.118 | -0.006 | 0.692 | 0.004 | 0.163 | -0.001 | 0.066 | 0.001 |
| Vocabulary | 0.759 | -0.065 | 0.122 | 0.001 | 0.403 | 0.001 | 0.806 | 0.001 | 0.882 | 0.001 | 0.389 | -0.001 | 0.220 | -0.008 | 0.064 | 0.031 | 0.601 | 0.001 | 0.449 | 0.001 |
| Word reading | 0.538 | 0.150 | 0.133 | 0.001 | 0.816 | 0.001 | 0.335 | 0.001 | 0.535 | 0.001 | 0.371 | -0.001 | 0.222 | -0.010 | 0.102 | 0.031 | 0.699 | 0.001 | 0.200 | 0.001 |
| FC: Functional connectivity, M: Male, F: Female, WA: White American, AA: African American, SBP: Systolic blood pressure, DBP: Diastolic blood pressure, WHM: White matter hyperintensities. | | | | | | | | | | | | | | | | | | | | |
